# Hypothalamic perifornical Urocortin-3 neurons modulate defensive responses to a potential threat stimulus

**DOI:** 10.1016/j.isci.2020.101908

**Published:** 2020-12-09

**Authors:** Noriko Horii-Hayashi, Kensaku Nomoto, Nozomi Endo, Akihiro Yamanaka, Takefumi Kikusui, Mayumi Nishi

**Affiliations:** 1 Department of Anatomy and Cell Biology, Nara Medical University, Kashihara, Nara 643-8521, Japan; 2 Companion Animal Research Laboratory, School of Veterinary Medicine, Azabu University, Sagamihara, Kanagawa 252–5201, Japan; 3 Department of Neuroscience II, Research Institute of Environmental Medicine, Nagoya University, Nagoya 464-8601, Japan; 4 Department of Physiology, Dokkyo Medical University, Mibu, Tochigi, 321-0293, Japan

**Keywords:** Biological Sciences, Neuroscience, Behavioral Neuroscience

## Abstract

Defensive behaviors are evolved responses to threat stimuli, and a potential threat elicits risk assessment (RA) behavior. However, neural mechanisms underlying RA behavior are hardly understood. Urocortin-3 (Ucn3) is a member of corticotropin-releasing factor peptide family and here, we report that Ucn3 neurons in the hypothalamic perifornical area (PeFA) are involved in RA of a novel object, a potential threat stimulus, in mice. Histological and *in vivo* fiber photometry studies revealed that the activity of PeFA Ucn3 neurons was associated with novel object investigation involving the stretch-attend posture, a behavioral marker for RA. Chemogenetic activation of these neurons increased RA and burying behaviors toward a novel object without affecting anxiety and corticosterone levels. Ablation of these neurons caused the abnormal behaviors of gnawing and direct contacts with novel objects, especially in a home-cage. These results suggest that PeFA Ucn3 neurons modulate defensive responses to a potential threat stimulus.

## Highlights

• The activity of PeFA Ucn3 neurons is associated with novel object investigation
• DREADD activation of PeFA Ucn3 neurons increases RA and burying of novel objects
• Ablation of PeFA Ucn3 neurons causes direct contact with a novel object
• PeFA Ucn3 neurons modulate defensive responses to novel objects

## Graphical Abstract

**Figure:**
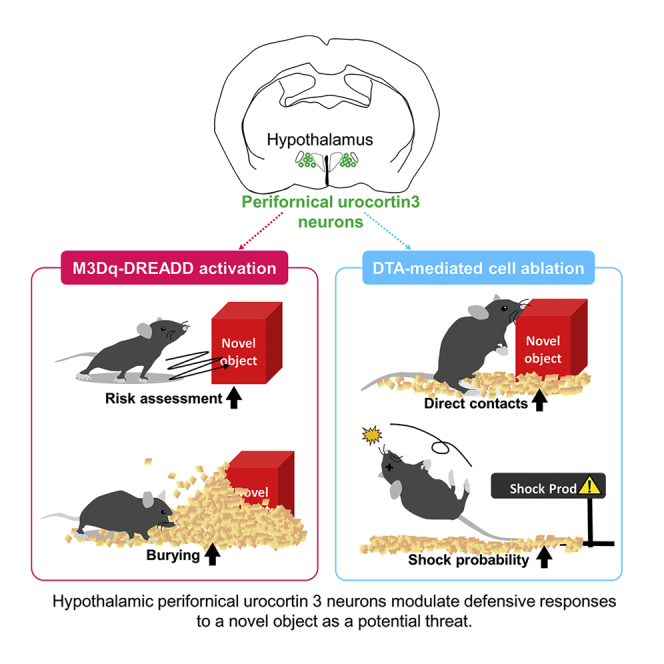

## Introduction

Defensive behaviors are a set of evolved responses to threat stimuli to avoid or reduce potential harm (Blanchard et al., 2010). Various forms of defensive behaviors ranging from risk assessment (RA) and freezing to flight and defensive attack (aggression) are elicited depending on threat imminence and contextual factors such as the existence of escape routes (Blanchard et al., 1991, 2001, 2011; Blanchard and Blanchard, 1969). Environmental cues indicating the unambiguous presence of an immediate threat give rise to fight or flight responses, whereas more diffuse and ambiguous cues elicit RA behavior (Grupe and Nitschke, 2013). The present study defines RA behavior as a pattern of activities involved in the detection and investigation of potential threat stimuli that lack information based on previous literature (Blanchard et al., 2010, 2011; McNaughton and Corr, 2018). Previous studies have identified brain regions responsible for specific defensive behaviors. The midbrain periaqueductal gray region is an essential part of the circuitry that elicits freezing and flight in response to threat (Carrive, 1993; Tovote et al., 2016), and the ventromedial hypothalamic nucleus plays a crucial role in the regulation of defensive aggression (Lin et al., 2011; Yang et al., 2013). However, neural mechanisms underlying the regulation of RA behavior are hardly understood.

Urocortin-3 is a member of the corticotropin-releasing factor (CRF) peptide family, which has been identified in humans and rodents (Hsu and Hsueh, 2001; Lewis et al., 2001). Ucn3 binds to the type 2 CRF receptor (CRFR2) with high affinity but not to the other known receptors within the family, such as CRFR1 (Hsu and Hsueh, 2001; Lewis et al., 2001). In the brain, Ucn3-expressing cells are observed in the hypothalamus, medial amygdala, parabrachial nucleus, and premamillary nucleus (Deussing et al., 2010; Kuperman et al., 2010; Lewis et al., 2001; Li et al., 2002). In the hypothalamus, Ucn3-expressing neurons are found in the median preoptic nucleus and rostral perifornical area (PeFA) lateral to the paraventricular nucleus (PVN) (Li et al., 2002). In rodents, PeFA Ucn3 neurons are considered to mainly project to the ventromedial hypothalamic nucleus (VMH) and the lateral septum (LS) (Autry et al., 2019; Chen et al., 2011; Kuperman et al., 2010).

Accumulating evidence has suggested that PeFA Ucn3 and its receptor, CRFR2, play an important role in energy homeostasis (Chen et al., 2012; Kuperman et al., 2010) and stress-related responses, including anxiety-like behaviors (Anthony et al., 2014; Kuperman et al., 2010; Venihaki et al., 2004). For example, overexpression of Ucn3 in the PeFA increased both the respiratory exchange ratio and heat production without affecting food intake in mice, in addition to elevating anxiety-like behaviors (Kuperman et al., 2010). Optogenetic activation of LS CRFR2 neurons, a target of PeFA Ucn3 neurons, promoted, whereas inhibition suppressed, anxiety-like behaviors (Anthony et al., 2014). However, differently from CRFR2-mutant mice (Bale et al., 2000; Bale and Vale, 2003; Coste et al., 2000), Ucn3-deficient mice did not show impairments in hypothalamic-pituitary-adrenal axis regulation and anxiety- or depression-related behaviors (Deussing et al., 2010). Furthermore, a more recent study has reported a different function of PeFA Ucn3 neurons from stress-related responses: the anterior portion of PeFA Ucn3 neurons (−0.1 to −0.5 mm to the bregma) is activated during infant-directed attack, and activation of these neurons elicit infant-directed neglect and aggression (Autry et al., 2019). Nevertheless, there are still no studies investigating the effects of activation and ablation/inhibition of PeFA Ucn3 neurons on anxiety-like behaviors. It has not been identified what kinds of stimuli apart from infant-directed aggression activate these neurons.

In the present study, we aimed to identify a stimulus activating PeFA Ucn3 neurons and elucidate the behavioral effects of activation/ablation of these neurons, including anxiety-like behaviors. To this end, we first investigated the reactivity of PeFA Ucn3 neurons to various forms of stimulus by c-Fos immunolabeling, a marker for activated neurons. Second, we performed fiber photometric analysis to identify behaviors associated with the activity of these neurons by Cre-dependent expression of GCaMP7s in PeFA Ucn3 neurons using *Ucn3-Cre* mice. Finally, we examined the behavioral effects of activation and ablation of PeFA Ucn3 neurons including anxiety-like behavior; the former made use of a pharmacogenetic method (designer receptors exclusively activated by designer drugs [DREADD]) and the latter used targeted cell ablation with diphtheria toxin subunit A (DTA) in *Ucn3-Cre* mice.

## Results

### Novel object stimulus activates PeFA Ucn3 neurons

**Figure 1:**
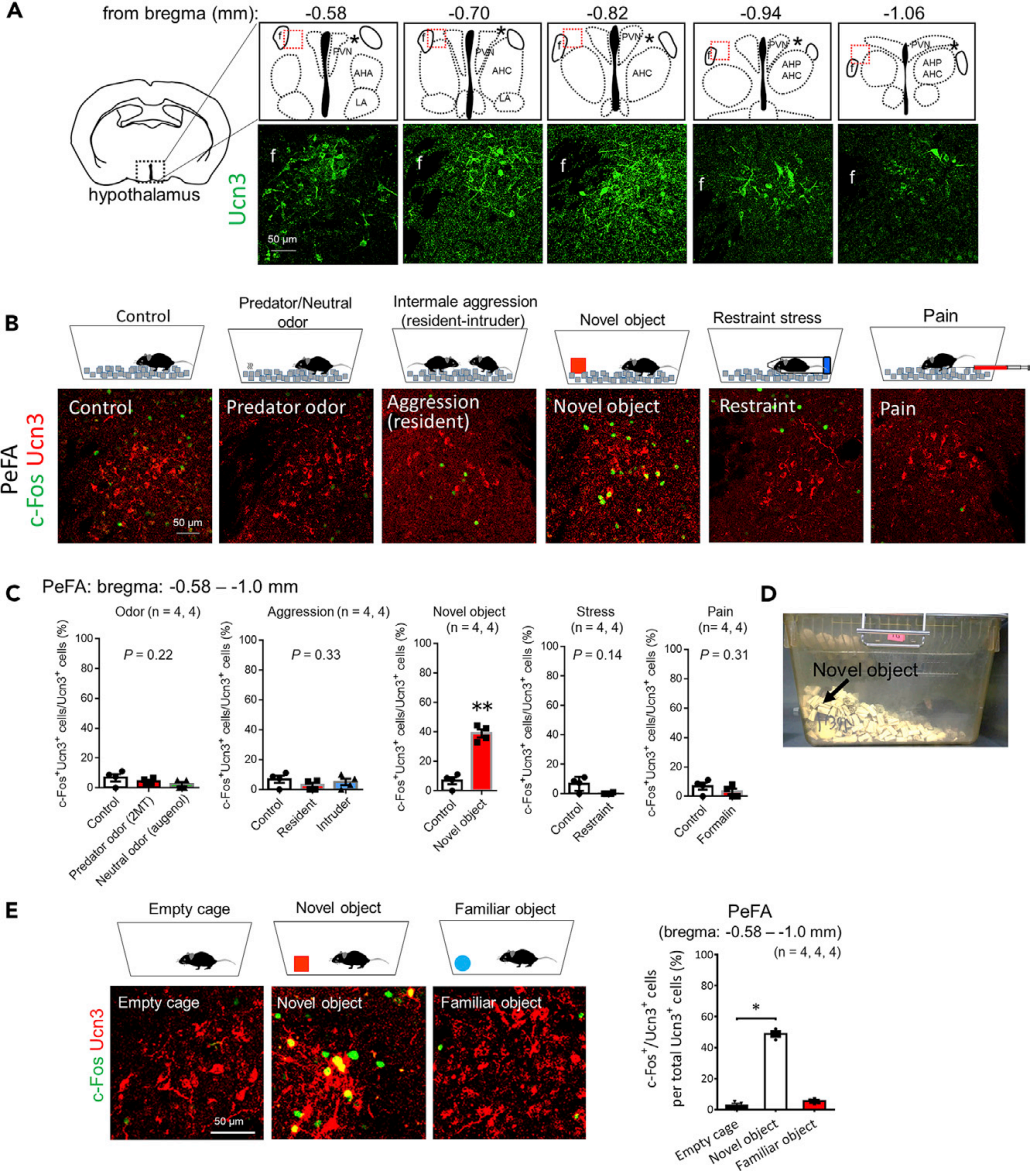
PeFA Ucn3 neurons are activated by a novel object stimulus (A) Schematic diagram showing the location of the PeFA (asterisks) and representative immunofluorescent images of Ucn3^+^ cells (green) in the PeFA indicated with red dotted lines. Scale bar: 50 μm. (B) Representative images of doubled-labeled sections of c-Fos (green) and Ucn3 (red) in the PeFA after exposure to stimuli in a home-cage: control (non-stimulated), predator odor (2MT), aggression (resident-intruder paradigm), novel object, restraint stress for 2 h, and pain (formalin injection into the hind paw). Scale bar: 50 μm. (C) The percentage of c-Fos and Ucn3 double-positive cells to Ucn3^+^ cells in the PeFA from −0.58 to −1.0 mm to the bregma. Data are represented as mean ± SEM (n = 4 animals in each group, odor: Kruskal-Wallis, p = 0.22; aggression: Kruskal-Wallis, p = 0.33; novel object, Mann-Whitney, U = 0, ∗∗p < 0.01; restraint stress, Mann-Whitney, U = 2.50, p = 0.14; pain, Mann-Whitney, U = 4.0, p= 0.31). (D) The appearance of a novel object buried with bedding material in the home-cage. (E) Representative images of doubled-labeled sections of c-Fos (green) and Ucn3 (red) in the PeFA after exposure to cage change (empty cage), novel object, and familiar object. The graph indicates the percentage of c-Fos and Ucn3 double-positive cells to Ucn3^+^ cells from −0.58 to −1.0 mm to the bregma. Data are represented as mean ± SEM (n = 4 animals in each group, Kruskal-Wallis p < 0.01, post hoc Dunn's test, empty cage versus novel object, ∗p < 0.05). Scale bar: 50 μm.

The present study focused on PeFA Ucn3 neurons beside the PVN, which were located −0.58 to −1.0 mm to the bregma, based on the mouse brain atlas (Figure 1A) (Franklin and Paxinos, 2007; George Paxinos, 2019). A previous study in rats indicated the co-expression of Enkephalin (Enk) in a subset of PeFA Ucn3 neurons that predominantly project to the LS (Chen et al., 2011). Because our previous mouse study showed that PeFA Enk neurons project to the LS (Horii-Hayashi et al., 2015), we verified the co-expression of these peptides in mice. Immunohistochemical results indicated that Ucn3^+^ neurons located −0.7 mm posterior to bregma co-expressed Enk (Figure S1A). Ucn3^+^/Enk^+^ nerve fibers were observed in the LS but not in the PVN or VMH (Figure S1B). These Ucn3^+^/Enk^+^ fibers formed perisomatic baskets around LS neurons (Figure S1C) as previously reported (Chen et al., 2011). To confirm that LS Enk fibers are derived from PeFA Ucn3 neurons, we ablated PeFA Ucn3 neurons using a Cre-dependent AAV for DTA expression (CMV-FLEX-mCherry/DTA) in *Ucn3-Cre* mice (Ucn3-DTA) or their wild-type littermates as a control (Figure S1D). Both Ucn3^+^ and Enk^+^ fibers in the LS were undetectable in Ucn3-neurons-ablated mice (Figure S1E), indicating that LS Enk fibers originate from PeFA Ucn3 neurons.

To investigate the types of stimuli that activate PeFA Ucn3 neurons, double labeling of c-Fos and Ucn3 was performed using mice exposed to one of the following stimuli or experiences in their home-cage: predator (2MT: 2-methyl-2-thiazoline) or neutral (eugenol) odor (Horio et al., 2019; Wang et al., 2018), intermale aggression by resident-intruder paradigm, novel object, restraint stress for 2 h, and pain by injecting formalin into the hind paw (Hunskaar and Hole, 1987). We confirmed that the mice smelled 2MT through observations of freezing behavior and increased c-Fos expression in the PVN (data not shown). Immunohistochemical results indicated that of all the stimuli, only the novel object stimulus increased c-Fos expression in PeFA Ucn3^+^ cells (Figure 1B). The percentage of c-Fos^+^/Ucn3^+^ cells to Ucn3^+^ cells was significantly higher than that of the non-stimulated control group (Figure 1C, control, 6.8 ± 2.4%; novel object, 39.0 ± 2.7%, Mann-Whitney, U = 0, ∗p < 0.05).

**Figure 2:**
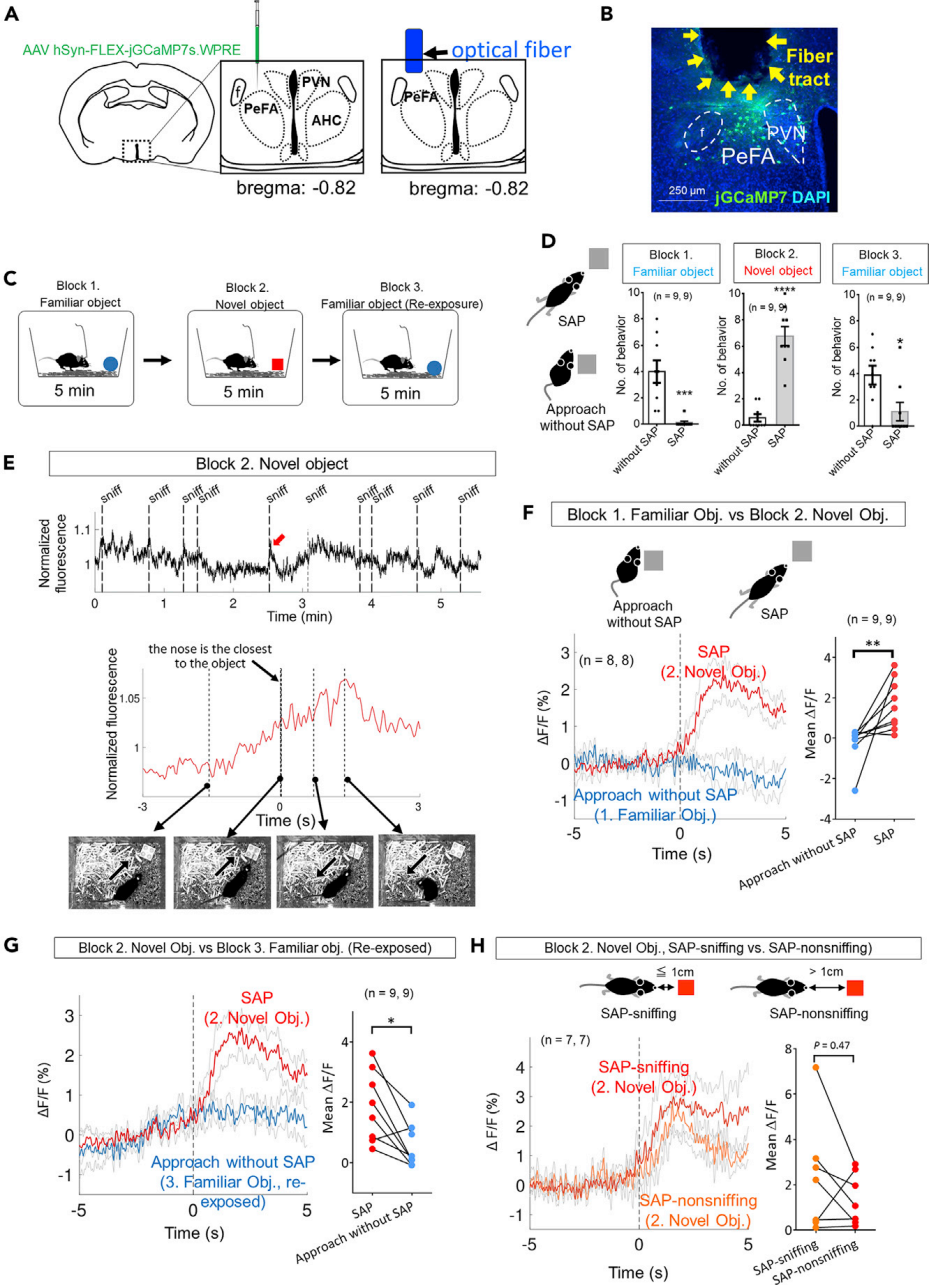
The activity of PeFA Ucn3 neurons is associated with novel object investigation involving SAP (A) Left: injection of Cre-dependent jGCaMP7s AAV into the PeFA at −0.82 mm to the bregma. Right: a schema showing optical fiber placement. (B) A representative image of the overlay of jGCaMP7 (green) and DAPI (blue) in the PeFA. Arrows indicate an optical fiber tract. Scale bar: 250 μm. (C) A schema showing the flow of experiments comprising three blocks. Mice were sequentially exposed to a familiar object (block 1), a novel object (block 2), and the same familiar object (block 3) for 5 min in each block in their home-cage. (D) Left: schematic diagrams showing the behaviors of SAP and approach without SAP. SAP: mice lower the back and stretch the neck toward an object with either standing still or moving forward without moving the hind paws; approach without SAP: mice stay near the object or touch it with the body or the forepaws. Right: graphs show the numbers of behaviors indicated in each block. Data are represented as mean ± SEM. Mann-Whitney test (n = 9 animals, ∗p < 0.05, ∗∗∗p < 0.001, ∗∗∗∗p < 0.0001). (E) Top: a representation of calcium-derived fluorescent signal during the block 2 experiment. Vertical dashed lines indicate the time when the mouse sniffed a novel object. Bottom: a magnified view of the fluorescent peak indicated with a red arrow in the top image. Time is adjusted to 0 when the mouse nose was closest to the novel object. Images show mouse behaviors at the time points indicated. The mouse approached the object while taking SAP (left), sniffed the object (second to the left), withdrew (second to the right), and retreated (right). Arrows represent the direction of animal movement. (F) Left: mean ΔF/F changes in block 1 and block 2 experiments. Time is adjusted to 0 when mice were closest to the object. Solid and thin gray lines represent the grand mean and SEM of calcium signals across animals, respectively (n = 9 animals). The number of events used for the calculation of the grand mean was, 5, 6, 8, 6, 6, 10, 3, 9, and 8 for the novel object block and 6, 2, 7, 8, 4, 1, 1, 4, and 3 for the familiar object block. Right: comparison between the grand mean ΔF/F during 5 s after the events. Wilcoxon signed-rank test, W = 43.0, ∗p < 0.01. (G) Left: mean ΔF/F changes in block 2 and block 3 experiments. Time is adjusted to 0 when mice are closest to the object. Solid and thin gray lines represent the grand mean and SEM of calcium signals across animals, respectively (n = 9 animals). The number of events used for the calculation of the grand mean was 5, 8, 6, 6, 10, 3, 9, and 8 for the novel object block and 6, 2, 7, 8, 4, 1, 1, 4, and 3 for the familiar object block (re-exposure). Right: comparison between the grand mean ΔF/F during 5 s following the events. Wilcoxon signed-rank test = −34.0, ∗p < 0.05. (H) Left: mean ΔF/F changes in block 2 experiment. SAP-related behaviors are classified into SAP-sniffing (a distance between the nose and the object is shorter than or equal to 1 cm) and SAP-nonsniffing (a distance between the nose and the object is longer than 1 cm). Time is adjusted to 0 when the mouse exhibited the most extended SAP. Solid and thin gray lines represent the grand mean and SEM of calcium signals across animals, respectively (n = 9 animals). The number of events used to calculate the grand mean was 2, 7, 1, 5, 2, 7, and 7 for SAP-sniffing and 4, 1, 5, 1, 8, 2, and 1 for SAP-nonsniffing. Right: comparison between the grand mean ΔF/F during 5 s following the events. Wilcoxon signed-rank test, W = −10.0, p = 0.47.

The novel object stimulus induced burying behavior in a home-cage (Figure 2D). Therefore, to examine whether burying behavior is directly associated with increased expression of c-Fos in Ucn3^+^ cells, a novel object stimulus was given in a cage without bedding material (empty cage) to block the behavioral expression of burying. A novel object stimulus in the cage induced c-Fos expression in Ucn3^+^ cells; the percentage of c-Fos^+^/Ucn3^+^ cells to Ucn3^+^ cells in the novel object group was significantly higher than that of the empty-cage (Figure 1E, Kruskal-Wallis, P < 0.01, post hoc Dunn's test, empty cage versus novel object, p < 0.05). These results indicated that a novel object stimulus activated PeFA Ucn3 neurons and that burying behavior was not directly associated with the activation of these neurons.

### Activity of PeFA Ucn3 neurons is associated with investigatory behaviors involving SAP toward a novel object

To investigate the activity dynamics of PeFA Ucn3 neurons during interaction with a novel object, we utilized an *in vivo* fiber photometry technique used to detect calcium dynamics. A Cre-dependent (FLEX) adeno-associated virus (AAV) encoding GCaMP7s, a calcium indicator, was injected into the PeFA of *Ucn3-Cre* mice at −0.82 mm to the bregma (Figure 2A). An optical fiber was then inserted immediately above the injection site (Figure 2B). The accuracy of the position of the inserted fiber and GCaMP7s expression were confirmed in all mice after the experiments (Figure S2A).

In a home-cage, the mice were sequentially exposed to a familiar object (block 1), a novel object (block 2), and the same familiar object again (block 3) for 5 min in each block (Figure 2C). In the novel object experiment (block 2), the mice approached and sniffed toward the object while performing the stretched-attend posture (SAP), which is a behavioral marker for RA and defined as a characteristic posture that animals lower the back, stretch the neck, and elongate the body toward potential danger either while standing still or moving forward (Blanchard et al., 1991, 2011; Holly et al., 2016; Molewijk et al., 1995; Reis et al., 2012; Roy and Chapillon, 2004). In contrast, mice showed very little SAP toward the familiar object in both block 1 and block 3 experiments (Figure 2D).

A representative trace of the calcium signals during the block 2 experiment is shown in Figure 2E, which indicates that several signal peaks were associated with sniffing behavior toward the novel object. A magnified peak and its associated behaviors showed that the calcium signal gradually increased as the mouse approached the object while performing SAP, had not reached a peak when the nose was closest to the object (Time 0), peaked when the mouse retreated from the object, and then gradually decreased (Figures 2E and Video S1). Comparison of the signal changes between block 1 and block 2 experiments revealed that the grand mean associated with SAP toward a novel object (block 2) was significantly higher than that associated with a familiar object without SAP (block 1) (Figure 2F; see Figure S2B for data of individual animals). Similar results were observed when comparing signals between the block 2 and block 3 experiments (Figure 2G; see Figure S2C for data of individual animals). These results indicate that the activity changes were not due to the order of the objects presented.

Video S1. Calcium dynamics during novel object investigation, related to figure 2

We next investigated whether sniffing or the distance between the nose and a novel object affects the activity of PeFA Ucn3 neurons. We classified SAP-related behaviors into two forms with reference to a previous study with slight modification (Gangadharan et al., 2016); SAP-sniffing and SAP-nonsniffing (Figure 2H). Based on these behavioral criteria, calcium signals during the block 2 experiment were analyzed, indicating that there was no significant difference between the two behavioral forms (Figure 2H; see Figure S2D for data of individual animals). These results indicated that the activity of PeFA Ucn3 neurons was associated with novel object investigation involving SAP behavior rather than sniffing itself.

### Activation of PeFA Ucn3 neurons by hM3Dq DREADD

**Figure 3:**
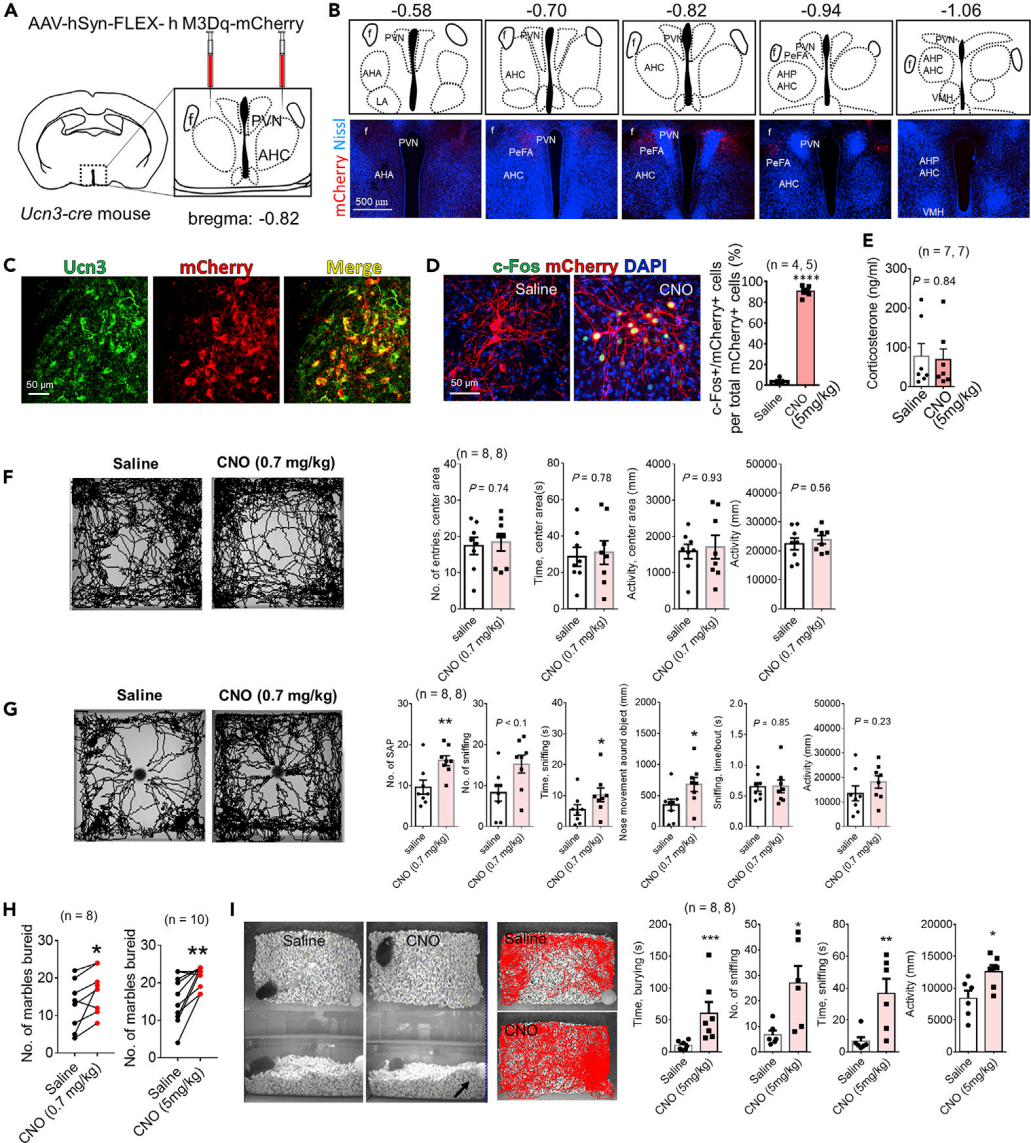
Activation of PeFA Ucn3 neurons promotes RA of a novel object and burying behavior (A) A schematic diagram showing Cre-dependent hM3Dq-mCherry AAV injection into the PeFA in *Ucn3-Cre* mice at −0.82 mm to the bregma. (B) Representative images of the overlay of mCherry (red) and Nissl (blue) in serial sections from AAV-injected *Ucn3-Cre* mice. Scale bar: 500 μm. (C) Representative fluorescent images showing Ucn3 (green) and mCherry (red) in the PeFA from AAV-injected mice. Scale bar: 50 μm. (D) Left: representative images showing the overlay of c-Fos (green) and mCherry (red) in the PeFA after saline or CNO injection (5 mg/kg mouse). Right: a graph shows the percentage of c-Fos and mCherry double-positive cells to mCherry + cells. Data are represented as mean ± SEM (n = 4, 5 animals, Mann-Whitney, U = 0, ∗∗∗∗p < 0.0001). Scale bar: 50 μm. (E) Plasma corticosterone levels 30 min after saline or CNO (5 mg/kg) injection. Data are represented as mean ± SEM (n = 7 animals, Mann-Whitney, U = 24, p = 0.84). (F) Open field test (n = 8, 8 animals). Representative images of body-center tracking in saline- or CNO (0.7 mg/kg mouse)-injected animals. Graphs show the number of entries into the center area (Mann-Whitney, U = 28.5, p = 0.74), duration of stay in the center area (Mann-Whitney, U = 29, p = 0.78), activity in the center area (Mann-Whitney, U = 31, p = 0.93), and activity in the whole field (Mann-Whitney, U = 26, p = 0.56). Data are represented as mean ± SEM. (G) Novel-object test (n = 8, 8 animals). Representative images of nose-point tracking in saline- or CNO (0.7 mg/kg mouse)-injected animals. Graphs show the number of SAP (Mann-Whitney, U = 8, ∗∗p < 0.01), the number of sniffing (Mann-Whitney, U = 13.5, p = 0.05), time engaged in sniffing (Mann-Whitney, U = 13, ∗p < 0.05), nose movement around the object (Mann-Whitney, U = 12, ∗p < 0.05), time per sniffing bout (Mann-Whitney, U = 30, p = 0.85), and activity in the whole field (Mann-Whitney, U = 20, p = 0.25). Data are represented as mean ± SEM. (H) Marble burying test. Graphs show the number of marbles buried in saline- or CNO-injected animals (left, CNO 0.7 mg/kg mouse, n = 8 animals; right, 5 mg/kg mouse, n = 10 animals). Saline versus CNO (0.7 mg/kg): Wilcoxon signed-rank test, W = 33.0, ∗p < 0.05. Saline versus CNO (5 mg/kg): p Wilcoxon signed-rank test, W = 43.0, ∗p < 0.01. (I) Single object burying test (n = 8, 8 animals). Left: the appearance of the test from overhead (upper line) and side-view (bottom line) cameras (left, saline; right, CNO, 5 mg/kg). An arrow indicates a buried object. Middle: representative images of nose-point tracking during single object burying test (top: saline, bottom: CNO). Right: graphs showing time engaged in burying (Mann-Whitney, U = 0, ∗∗∗p < 0.001), the number of sniffing (Mann-Whitney, U = 3, ∗p < 0.05), time engaged in sniffing (Mann-Whitney, U = 2, ∗∗p < 0.01), and activity in the whole field (Mann-Whitney, U = 50, ∗p < 0.05). Data are represented as mean ± SEM

To investigate behavioral effects of activation of PeFA Ucn3 neurons, we utilized the technique of hM3Dq-based DREADD by injecting Cre-dependent AAV encoding hM3Dq-mCherry (200‒250 nL) into the PeFA at −0.82 mm to the bregma in *Ucn3-Cre* mice (Figure 3A). In these mice, mCherry^+^ cells were mainly observed between the fornix and PVN from −0.58 to −0.94 mm to the bregma (Figure 3B). The majority of mCherry^+^ cells were immunoreactive for Ucn3 (Figure 3C). Intraperitoneal injection of clozapine N-oxide (CNO) induced c-Fos expression in 90.4 ± 2.0% of mCherry^+^ cells (n = 5), whereas saline injection induced c-Fos in 2.62 ± 1.2% of mCherry^+^ cells (Figure 3D, n = 4, 5, Mann-Whitney, U = 0, p < 0.0001). Plasma corticosterone levels 30 min after saline or CNO injection were comparable between the two groups (Figure 3E, Mann-Whitney, U = 24, p = 0.84).

### Activation of PeFA Ucn3 does not affect anxiety-like behaviors, but promotes SAP and sniffing during the novel object test

The open field test followed by the novel object test was performed to evaluate anxiety-like behaviors, in accordance with previous studies (Anthony et al., 2014; Dietrich et al., 2017). Although two different doses of CNO, 0.7 mg/kg (Figure 3) or 5.0 mg/kg (Figure S3), were tested, neither dose affected the number of entries into the center area, duration and activity in the center area, and activity in a whole field in the open field test (Figures 3F and S3B). Conversely, the novel object test revealed that both doses of CNO significantly increased the number of SAP, time consumed for sniffing (a distance between the nose and the object <1 cm), and nose movement 1 cm around the object, compared with those of the individual saline groups (Figures 3G and S3C). In addition, although we performed light-dark box test and elevated plus-maze test for assessing anxiety-like behavior with reference to previous studies (Anthony et al., 2014; Komada et al., 2008; Takao and Miyakawa, 2006), there were no significant differences in all behavioral parameters measured between saline and CNO (5 mg/kg) groups (Figures S3D and S3E).

When a control vector, AAV: hSyn-FLEX-mCherry, was injected into the PeFA with the same coordinate (Figure S4A), CNO administration (5 mg/kg) had no effect on behaviors in both the open-field (Figure S4B) and novel object test (Figure S4C), indicating that CNO-induced behavioral effects observed in the novel object test were caused via hM3Dq. These results indicated that activation of PeFA Ucn3 neurons did not affect anxiety-like behaviors, whereas it increased SAP and sniffing behaviors toward a novel object, which were consistent with the results of c-Fos expression (Figure 1) and fiber photometric (Figure 2) experiments.

### Activation of PeFA Ucn3 neurons promotes burying behavior

The marble-burying test is widely used to examine burying activity in mice and is also applied to evaluate anxiolytic and anticompulsive drug actions (Deacon, 2006; Thomas et al., 2009). CNO treatment with both doses (0.7 mg/kg and 5 mg/kg) significantly increased the number of marbles buried compared with saline treatment (Figure 3H). We further measured burying activity using a single novel object, indicating that CNO administration (5 mg/kg) significantly increased burying behavior in addition to increased sniffing and locomotor activity in the field (Figures 3I and Video S2). These results indicated that activation of PeFA Ucn3 neurons promoted burying behavior.

Video S2. Increased burying in PeFA Ucn3 neurons-activated mice, related to figure 3

The location of mCherry^+^ cells and their Ucn3 expression were confirmed after behavioral testing. Results are shown in Figures S5 and S6 from 16 animals used for 0.7 mg/kg CNO or saline; mCherry^+^ cells were distributed from −0.58 to −0.94 mm to the bregma (Figure S5) and 87.0 ± 1.68% of mCherry^+^ cells were immunoreactive for Ucn3 (Figure S6, n = 16).

### Ablation of PeFA Ucn3 neurons has no effects on SAP and burying behaviors

**Figure 4:**
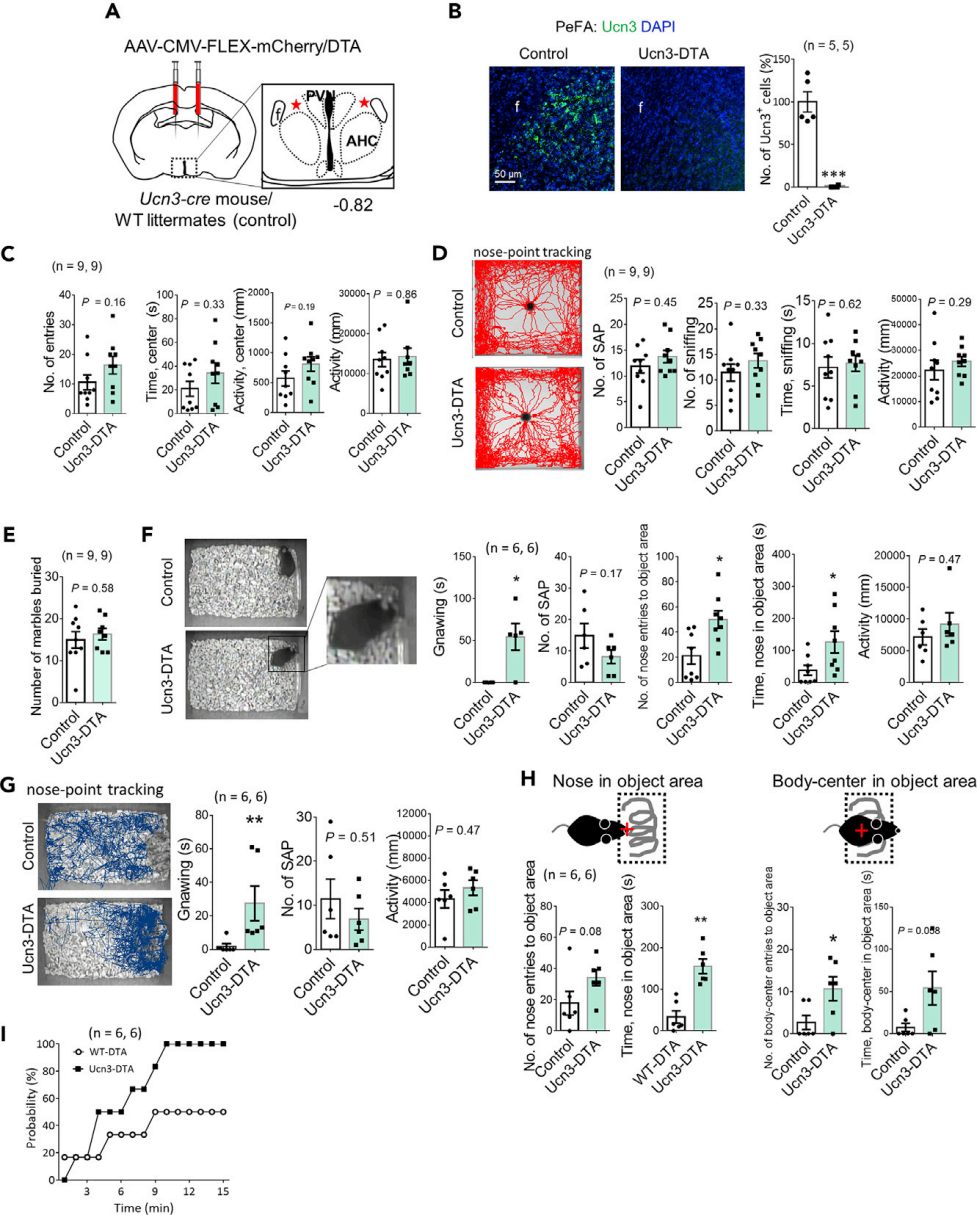
Ablation of PeFA Ucn3 neurons causes close contacts with novel objects particularly in a home-cage (A) A schematic representation of injection of Cre-dependent mCherry/DTA AAV into the PeFA at −0.82 mm to the bregma of *Ucn3-Cre* mice (Ucn3-DTA) or their wild-type littermates (control). (B) Representative images showing Ucn3^+^ cells (green) in the PeFA in control and Ucn3-DTA mice. A graph shows the percentage of the number of Ucn3^+^ cells in the PeFA when the values of control animals being 100%. (Mann-Whitney, U = 0, ∗p < 0.05, n = 5 animals). Scale bar: 50 μm. (C) Open field test (n = 9, 9 animals). Graphs show the number of entries into the center area (Mann-Whitney, U = 24.5, p = 0.16), duration of stay in the center area (Mann-Whitney, U = 29, p = 0.33), activity in the center area (Mann-Whitney, U = 25, p = 0.19), and activity in the whole field (Mann-Whitney, U = 346, p = 0.86). Data are represented as mean ± SEM. (D) Novel-object test (n = 9, 9 animals). Representative images of nose-point tracking in control (up) and Ucn3-DTA (bottom) mice. Graphs show the number of SAP (Mann-Whitney, U = 31.5, p = 0.45), the number of sniffing (Mann-Whitney, U = 29, p = 0.33), time engaged in sniffing (Mann-Whitney, U = 34.5, p = 0.62), and activity in the field (Mann-Whitney, U = 28, p = 0.29). Data are represented as mean ± SEM. (E) Marble burying test (n = 9, 9 animals). Graphs show the number of marbles buried (mean ± SEM, Mann-Whitney, U = 33, p = 0.79). (F) Novel-object test in a home-cage (plastic tube, n = 6, 6 animals). The appearance of the test (up: control, bottom: Ucn3-DTA). An enlarged view of a boxed area shows gnawing behavior. Graphs show time engaged in gnawing the object (Mann-Whitney, U = 3, ∗p < 0.05), the number of SAP (Mann-Whitney, U = 10.5, p = 0.25), the number of nose entries to the object area (1 cm around the object) (Mann-Whitney, U = 9.5, ∗p < 0.05), time that the nose is in the object area (Mann-Whitney, U = 11, ∗p < 0.05), and activity in the field (Mann-Whitney, U = 13, p = 0.47). Data are represented as mean ± SEM. (G) Novel-object test in the home-cage (a coiled barbwire, n = 6, 6 animals). Representative images of nose-point tracking in control (up) and Ucn3-DTA (bottom) mice. Graphs show time engaged in gnawing the object (Mann-Whitney, U = 0, ∗∗p < 0.01), the number of SAP (Mann-Whitney, U = 13.5, p = 0.51), and activity in the field (Mann-Whitney, U = 13, p = 0.47). Data are represented as mean ± SEM. (H) The illustrations indicate a nose-point (left) or body-center (right) tracking in the object area (1 cm around the object). Data are represented as mean ± SEM. The number of nose entries to the object area (Mann-Whitney, U = 7, p= 0.08), time that the nose was in the object area (Mann-Whitney, U = 0, ∗∗p < 0.01), the number of body-center entries to the object area (Mann-Whitney, U = 6, p < 0.05), and time that the body-center was in the object area (Mann-Whitney, U = 6, p = 0.058). (I) A graph showing the probability that mice receive electric shock in the home-cage (n = 6, 6, Wilcoxon signed-rank test, ∗∗∗p < 0.001).

We next investigated the effects of ablation of PeFA Ucn3 neurons on anxiety-like behaviors, responses to a novel object, and burying behavior. As described in Figure S1D, DTA-induced targeted cell ablation was performed using *Ucn3-Cre* mice (Figure 4A). Almost all PeFA Ucn3^+^ cells had disappeared in the PeFA of Ucn3-DTA mice but not in control mice (Figure 4B, control: 100 ± 11.9%; Ucn3-DTA: 0.89 ± 0.61%, Mann-Whitney, U = 0, p < 0.01). We performed the open field test (Figure 4C), novel object test (Figure 4D), and marble-burying test (Figure 4E); there were no significant differences in the behaviors on any tests, indicating that ablation of PeFA Ucn3 neurons did not affect either anxiety levels or behavioral expressions of SAP, sniffing, and burying. After behavioral testing, we histologically confirmed the ablation of Ucn3^+^ cells in the PeFA from all mice tested (data not shown).

### PeFA Ucn3-neuron-ablating mice make direct contact with a novel object, particularly in a home-cage

We next investigated the behavioral responses to a novel object in a home-cage using Ucn3-DTA mice. When a novel object (plastic tube) was put in the home-cage, the number of SAP toward the object and activity in the cage were comparable between control and Ucn3-DTA groups, whereas Ucn3-DTA mice gnawed and persistently sniffed the object (Figure 4F). When a different object, a coiled barbwire, was used as a novel object, similar results were obtained (Figure 4G and see Video S3). Furthermore, we performed an analysis using body-center tracking in the object area (1 cm around the object) in addition to that of the nose-point (Figure 4H). The results indicated that the time that the nose was in the object area as well as the number of body-center entries in the same area were significantly increased in Ucn3-DTA mice compared with respective controls (Figure 4H). Both the number of nose entries into the object area and the time that the body-center was in the same area also showed an increased tendency in Ucn3-DTA mice compared with respective controls (Figure 4H). These results indicate much closer physical interactions with a novel object in Ucn3-DTA mice compared with control animals in a home-cage.

Video S3. Increased direct contacts with a novel object in PeFA Ucn3 neurons-ablated mice, related to figure 4

To examine whether mice actually touch novel objects, we used a shock prod that releases an electric current only when a moist object, such as the nose or mouth, touches the prod. The probability that mice receive an electric shock in Ucn3-DTA group reached 100% within 15 min, whereas the probability of control mice reached a maximum of 50% within the same time window (Figure 4I). These results indicated that ablation of PeFA Ucn3 neurons increased direct contacts with a novel object, particularly in the home-cage.

Because Ucn3 and CRFR2 have been shown to modulate metabolism (Chen et al., 2010; Kuperman et al., 2010), we measured body weight, its changes before and after viral injection, food intake, and plasma corticosterone levels in Ucn3-DTA mice. However, there were no significant differences in any measurements between the control and Ucn3-DTA groups (Figure S7).

## Discussion

The purpose of the present study was to identify stimuli that activate PeFA Ucn3 neurons and elucidate the behavioral effects of activation and ablation of these neurons, including anxiety-like behaviors. Here, we first demonstrated that the activity of PeFA Ucn3 neurons was increased by a novel object stimulus and associated with SAP behavior during novel object investigation. Second, activation of PeFA Ucn3 neurons had no effects on anxiety-like behaviors at least in non-stressed animals as measured by open field, light-dark box, elevated plus-maze tests, whereas it increased SAP and sniffing toward a novel object and burying behavior. Third, ablation of PeFA Ucn3 neurons had no effects on the behavioral expression of SAP and burying, whereas it induced much close and direct contacts with a novel object including gnawing and persistent sniffing, particularly in the home-cage. These abnormalities led to an increased risk of mice receiving an electric shock when the object was an electrified prod. Finally, neither activation nor ablation of these neurons affected plasma corticosterone level. These results indicate that PeFA Ucn3 neurons modulate RA toward novel objects and likely contribute to keep an appropriate distance from the object to avoid the risk brought by potential threats particularly in the habituated environment of a home-cage.

### Neither activation nor ablation of PeFA Ucn3 neurons affects anxiety and plasma corticosterone levels

It is generally believed that CRF/CRFR1 signaling promotes stress responses and anxiety-like behavior, whereas Ucns/CRFR2 mediates stress recovery and the restoration of homeostasis; however, recent studies reveal that this view is overly simplistic and CRFRs signaling are brain-region- and cell-type-specific (Henckens et al., 2016). Importantly, previous studies on Ucn3 neurons have utilized vastly different experimental approaches, animal conditions, and evaluation methods. Therefore, it's impossible to conclude whether Ucn3's function is anxiogenic or anxiolytic based on any one study. However, systemic or whole brain manipulation studies such as CRFR2 knockout (Bale et al., 2000; Bale and Vale, 2003) and cerebroventricular injection of Ucn3 (Telegdy and Adamik, 2013; Telegdy et al., 2011; Venihaki et al., 2004) seem to lead to the conclusion that Ucn3/CRFR2 signaling has anxiolytic effects. On the other hand, the results from Ucn3/CRFR2 studies focusing on the PeFA or LS, are more complicated. That is, they fall under one of the following: anxiogenic in non-stressed conditions (Anthony et al., 2014; Kuperman et al., 2010); anxiogenic in stressed conditions (Henry et al., 2006; Radulovic et al., 1999); or having no effect, at least in non-stressed conditions (Henry et al., 2006). The present study is included in the last group.

Although the reason for these inconsistent findings on the functions of LS Ucn3/CRFR2 signaling in the context of anxiety is not entirely clear, there are at least three possibilities. First, as demonstrated by Henry et al. (2006) and Radulovic et al. (1999), the behavioral effects of Ucn3/CRFR2 in the LS are affected by stress, thus activation of PeFA Ucn3 neurons in stressed animals may increase anxiety-like behavior, an important experiment for future study. Second, the behavioral effects of Ucn3/CRFR2 might depend on increased expression of Ucn3, as there are reports demonstrating that stress increases Ucn3 mRNA levels (Venihaki et al., 2004) and increased anxiety-like behavior has been reported after overexpression of Ucn3 in PeFA neurons (Kuperman et al., 2010). The third possibility centers around the involvement of Enk signaling. All previous studies manipulated either Ucn3 or CRFR2 in the PeFA or the LS, whereas the present DREADD activation likely promoted Enk release in the LS simultaneous with Ucn3, because a portion of PeFA Ucn3 neurons co-express Enk and these co-expressing neurons predominantly project to the LS. Optogenetic activation of LS CRFR2 neurons increases anxiety-like behavior in both non-stressed and stressed animals (Anthony et al., 2014). However, PeFA-neuron-derived inputs are considered to stimulate LS μ-opioid receptor-expressing neurons as well as CRFR2-expressing neurons. The complex effects of multiple neuropeptides might cause qualitative behavioral changes such as a transition from avoidance of a novel object to RA, because negative valence possibly causes various forms of defensive behavior including avoidance, immobility, and RA. Since RA contains emotional conflict between internal approach and withdrawal tendencies (McNaughton and Corr, 2018), multiple neuropeptides might be related to this behavior. Therefore, an important future direction is to investigate the complex effects of Ucn3 and Enk in the LS on behaviors and to clarify whether CRHR2 and the μ-opioid receptor are expressed by the same or different neurons in the LS.

### Activation of PeFA Ucn3 neurons increases active forms of defensive behavior toward a novel object

**Figure 5:**
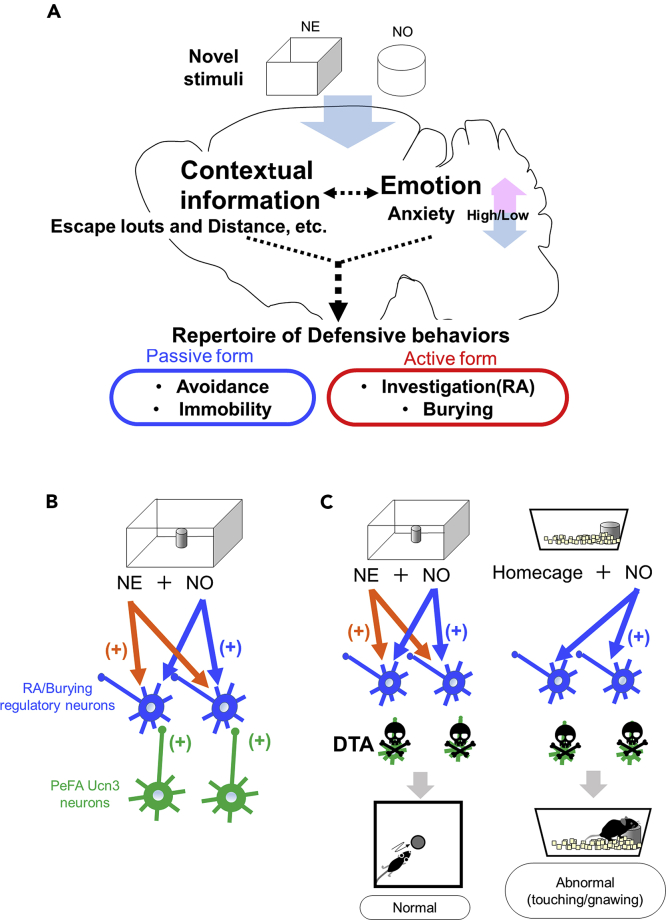
Schematic diagrams showing the function of PeFA Ucn3 neurons and a possible circuit model for RA/burying behavior (A) The schema indicates a simplified process of the expression of defensive behaviors in response to novel stimuli. A novel environment (NE) and novel object (NO) stimulate neural circuits involved in the processing of contextual information and emotional control, and one behavioral form is selected from the repertoire of defensive behaviors. Defensive behaviors can be divided into two forms, passive (avoidance and immobility) and active (investigation/RA and burying). PeFA Ucn3 neurons are involved in the latter form. (B) A possible circuit model for RA/burying behavior: Novel stimuli (NE and NO) stimulate RA/burying regulatory neurons that receive inputs from PeFA Ucn3 neurons. (C) A possible mechanism explaining different behavioral responses to a novel object in a novel environment and home-cage in Ucn3-neurons-ablated mice.

Ethologically, both RA and burying behaviors in rodents are categorized as defensive behavior (Blanchard et al., 1991, 2011; De Boer and Koolhaas, 2003; Koolhaas et al., 1999). A simplified model indicating the process involved in the expression of defensive behaviors elicited by novel stimuli (potential threats) is shown in Figure 5A, based on previous literature (Blanchard et al., 2010, 2011; Calhoon and Tye, 2015; Gangadharan et al., 2016; McGregor et al., 2020). Novel stimuli, including environments and objects, stimulate neural circuits involved in the processing of contextual information and emotional control eliciting a suitable behavioral response selected from the repertoire of defensive behaviors (Figure 5A). Although the LS, a projection target of PeFA Ucn3 neurons, is a regulatory center for anxiety (Anthony et al., 2014), our results indicated that neither activation nor ablation of PeFA Ucn3 neurons changed anxiety-like behavior. Thus, RA increased by activation of these neurons is not probably caused by the alteration of anxiety.

There is an interesting hypothesis that suggests defensive behaviors can be divided into two categories, active and passive (Coppens et al., 2010; de Boer et al., 2017; Koolhaas et al., 1999). It is important to note that this hypothesis was generated largely through behavioral observations from the shock-prod test in rats (Coppens et al., 2010). However, this categorization seems to be applicable to a novel object and thus potentially useful for understanding the function of PeFA Ucn3 neurons. According to this idea, avoiding the shock prod and immobility (freezing) are classified as a passive form of defensive behavior, whereas burying is an active form of defensive behavior (Figure 5A). Therefore, in terms of the proximity and approach to objects, RA behavior can be classified as an active form of defensive behavior (Figure 5A). Based on these considerations, PeFA Ucn3 neurons increase active forms of defensive behavior toward a potential threat. An interesting point is that although RA and burying are ostensibly different behavioral outputs, both behaviors are promoted by the same neurons (i.e. PeFA Ucn3 neurons). This fact is consistent with the thought that defensive burying has an RA component, which was described by Pinel et al. (1994) more than 25 years ago through defensive burying experiments performed in total darkness (Pinel et al., 1994). Our results indirectly support this idea given that the same neurons promote these two kinds of behaviors.

Another important point is that the ablation of these neurons had no effects on the behavioral expression of RA and burying, which suggests that a critical center for regulation of these behaviors exist somewhere else in the brain. We believe that the most probable region is the septum including the LS, because a large number of studies have reported that a septal lesion decreases defensive burying (De Boer and Koolhaas, 2003) and the LS is a target of PeFA Ucn3 neurons. It is unclear why PeFA Ucn3 neurons-ablated mice showed increased direct contacts with a novel object particularly in a home-cage, albeit no behavioral impairments in the novel object test performed in novel environment. It is possible that RA/Burying regulatory neurons that receive inputs from PeFA Ucn3 neurons are also innervated from other neurons responding to novel environment and novel object stimuli (Figure 5B). This hypothesis is based on the evidence that exploration of novel objects and novel environments are regulated by different neural networks within the septo-hippocampal circuits (Fuhrmann et al., 2015; Gangadharan et al., 2016). Second, previous studies have found that PeFA Enk neurons (i.e. Ucn3 neurons) are not GABAergic (Horii-Hayashi et al., 2015; Varoqueaux and Leranth, 1997), form asymmetric synapses with LS neurons, and LS neurons surrounded by PeFA-derived Enk fibers receive inputs from hippocampal excitatory neurons (Varoqueaux and Leranth, 1997).

Based on the above hypothesis, when the novel object test is performed in a novel environment, even though PeFA Ucn3 neurons are ablated by DTA, novel-environment- and novel-object-responding neurons stimulate RA/Burying regulatory neurons (Figure 5C, left). However, when a novel object is put in a home-cage, inputs to RA/Burying regulatory neurons are limited from novel object-related neurons (Figure 5C). Quantitative and/or qualitative changes in inputs to RA/burying regulatory neurons might induce abnormal and careless-like behaviors such as direct touching and gnawing of a novel object particularly in a home-cage. To understand the neural mechanism underlying RA/burying behavior, further studies focusing on the LS and the hippocampus are required.

### The activity of PeFA Ucn3 neurons during RA of a novel object

The results of c-Fos expression experiments indicate that stressful events, aggression, predator odor, and pain, are not likely to be a direct factor to activate PeFA Ucn3 neurons, and a novel object stimulus, a form of potential threats, can activate these neurons. Although many studies demonstrate that central Ucn3 and CRFR2 are involved in stress-related responses (Chen et al., 2012; Henckens et al., 2016; Henry et al., 2006; Kuperman et al., 2010; Radulovic et al., 1999), this study found another significant role of PeFA Ucn3 neurons under non-stressed conditions.

Considering the fiber photometry result that the peak of calcium signal was observed when the mice withdrew from a novel object, increased physical contacts and the failure of keeping an appropriate distance from a novel object in Ucn3-neurons-ablated mice may be caused by impairments in withdrawing from the object. Through such fine behavioral modulations, PeFA Ucn3 neurons probably contribute to avoiding the risk brought by potential threats during RA and play an important role in animal survival.

There is a report demonstrating that PeFA Ucn3 neurons, particularly a rostral population (more rostral than −0.5 mm to the bregma), not caudal, were activated by infant-direct aggression (Autry et al., 2019). Based on the anatomical coordinates, the population of PeFA Ucn3 neurons activated by infant-direct aggression may differ from those activated by a novel object stimulus. Because PeFA Ucn3 neurons project to multiple brain regions, the LS and VMH (Autry et al., 2019; Chen et al., 2011), these neurons might have different functions depending on anatomical locations.

### Limitations of the study

First, although this study found that novel objects activate PeFA Ucn3 neurons, the stimuli used for our experiments was very limited. Thus, the present results cannot deny the possibility that PeFA Ucn3 neurons could be activated by other kinds of stimuli other than a novel object. Particularly, although predator odor is known to elicit RA behavior (Kalynchuk et al., 2004), it is unclear whether mice exhibited RA behavior even once during 2MT exposure, because we confirmed 2MT effects through freezing behavior and increased c-Fos expression in the PVN. In addition to predator odor-induced RA, rodents exhibit SAP during the social interaction (Henriques-Alves and Queiroz, 2015), elevated plus-maze (Holly et al., 2016), and canopy SAP (Grewal et al., 1997) tests. Investigation of the activity of PeFA Ucn3 neurons during these tests is an important subject for future research. Second, we used the elevated plus-maze test to measure anxiety-like behavior, not to measure RA behavior (SAP). Thus, future studies coupling SAP-related behavioral tests with PeFA Ucn3 neurons' manipulations will be important. Third, behavioral tests were performed in the light phase due to our research environment. Therefore, the behavioral results might vary based on when the tests are performed, particularly if performed in the dark phase. Finally, all of our experiments were performed using male mice, thus future studies are needed to investigate potential sex differences.

### Resource availability

#### Lead contact

Further information and requests for resources and reagents should be directed to and will be fulfilled by the Lead Contact, Noriko Horii-Hayashi (hayashi@naramed-u.ac.jp).

#### Materials availability

The study did not generate new unique agents.

#### Data and code availability

This study did not generate/analyze datasets and code.

## Methods

All methods can be found in the accompanying Transparent Methods supplemental file.

## Supplemental information

Document S1. Transparent Methods and Figures S1–S7
