# Supplementary material for: Hypothalamic perifornical Urocortin-3 neurons modulate defensive responses to a potential threat stimulus
Source: iScience. 2020 Dec 9;24(1):101908. doi: 10.1016/j.isci.2020.101908 (PMC7770982; doi:10.1016/j.isci.2020.101908)
Supplement: Document S1. Transparent Methods and Figures S1–S7 [file mmc1.pdf]

iScience, Volume *24*

Supplemental Information

Hypothalamic perifornical Urocortin-3 neurons modulate defensive responses to a potential threat stimulus

Noriko Horii-Hayashi, Kensaku Nomoto, Nozomi Endo, Akihiro Yamanaka, Takefumi Kikusui, and Mayumi Nishi

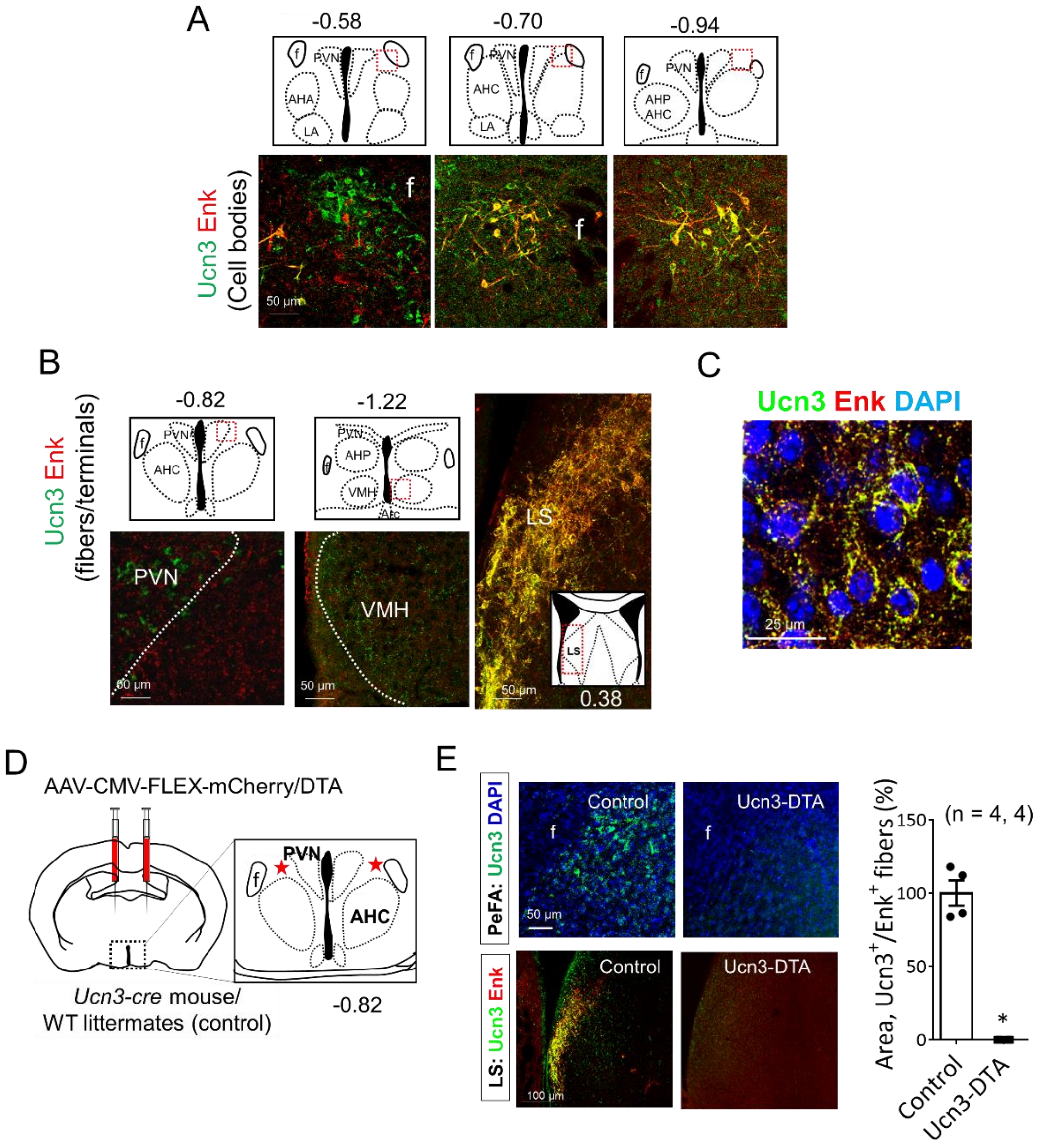

Figure S1. PeFA Ucn3/Enk co-expressing neurons project to the LS. Related to Figure 1.

(A)Representative images of double-labeling of Ucn3 (green) and Enk (red) in the PeFA. The fluorescent pictures are taken from squared regions indicated by red dotted lines on the brain atlas. Scale bar = 50 μm.

(B)Double-labeled images of Ucn3 (green) and Enk (red) in nerve fibers from the PVN (left), VMH (middle), and LS (right). The pictures are taken from the brain regions indicated with red dotted lines on the brain atlas. Scale bars = 50 μm.

(C)A higher magnification view of Ucn3 (green)- and Enk(red)-double-labeled fibers surrounding LS neurons (blue, DAPI). Scale bar = 100 μm.

(D)A schematic representation of injection of Cre-dependent mCherry/DTA AAV into the PeFA at -0.82 mm to the bregma of Ucn3-Cre mice (Ucn3-DTA) or their wildtype littermates (control).

(E)Representative images showing the ablation of $Ucn3^{+}$ cells (green) cells in the PeFA from Ucn3-DTA mice (top). Double labeling of Ucn3 (green) and Enk (red) in control and Ucn3-DTA mice showing the disappearance of $Ucn3^{+}/Enk^{+}$ fibers in the LS from Ucn3-DTA mice (bottom. The graph indicating the relative value of the percentage of $Ucn3^{+}/Enk^{+}$ area in the LS when the control group being 100%. Mann-Whitney test (n = 4 animals, $P < 0.05$). Scale bars = 50 μm (PeFA) and 100 μm (LS).

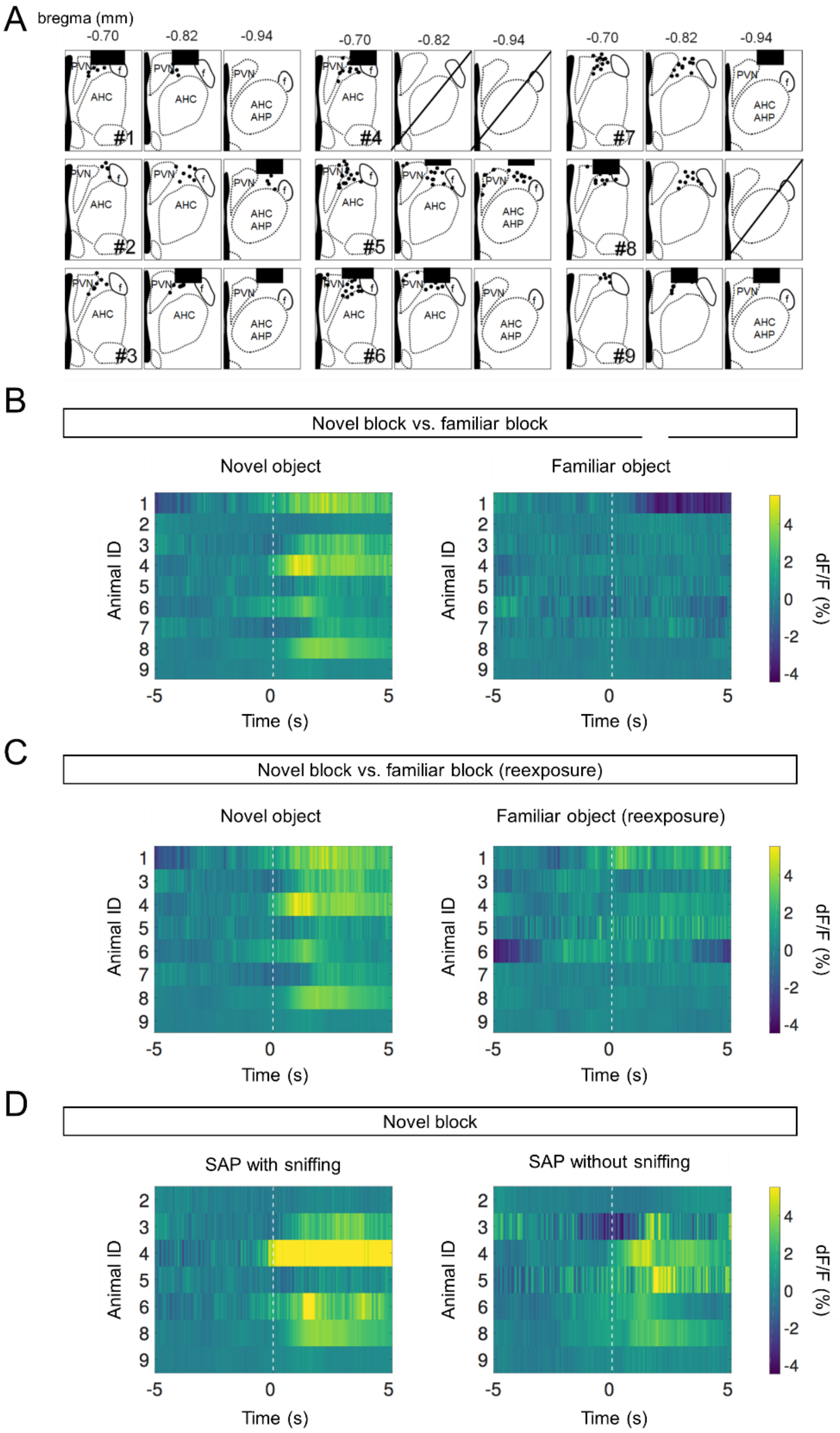

Figure S2. Fiber placement, GCaMP6 expression and photometric signals from individual animals. Related to Figure 2.

(A)Illustrations indicating the location of GCaMP6-expressing cells (black dots) and the placement of the optical fiber (black rectangles). Slashed images indicate non-analyzed sections due to damage.

(B – D) Color indicates ΔF/F. Each row represents the data obtained from one animal. Time was adjusted to 0 when the mouse was closest to the object. B: Block 2 (novel object) vs. Block 1 (familiar object). C: Block 2 (novel object) vs. Block 3 (familiar object, re-exposure). D: Block 2 (novel object), SAP-sniffing vs. SAP-nonsniffing

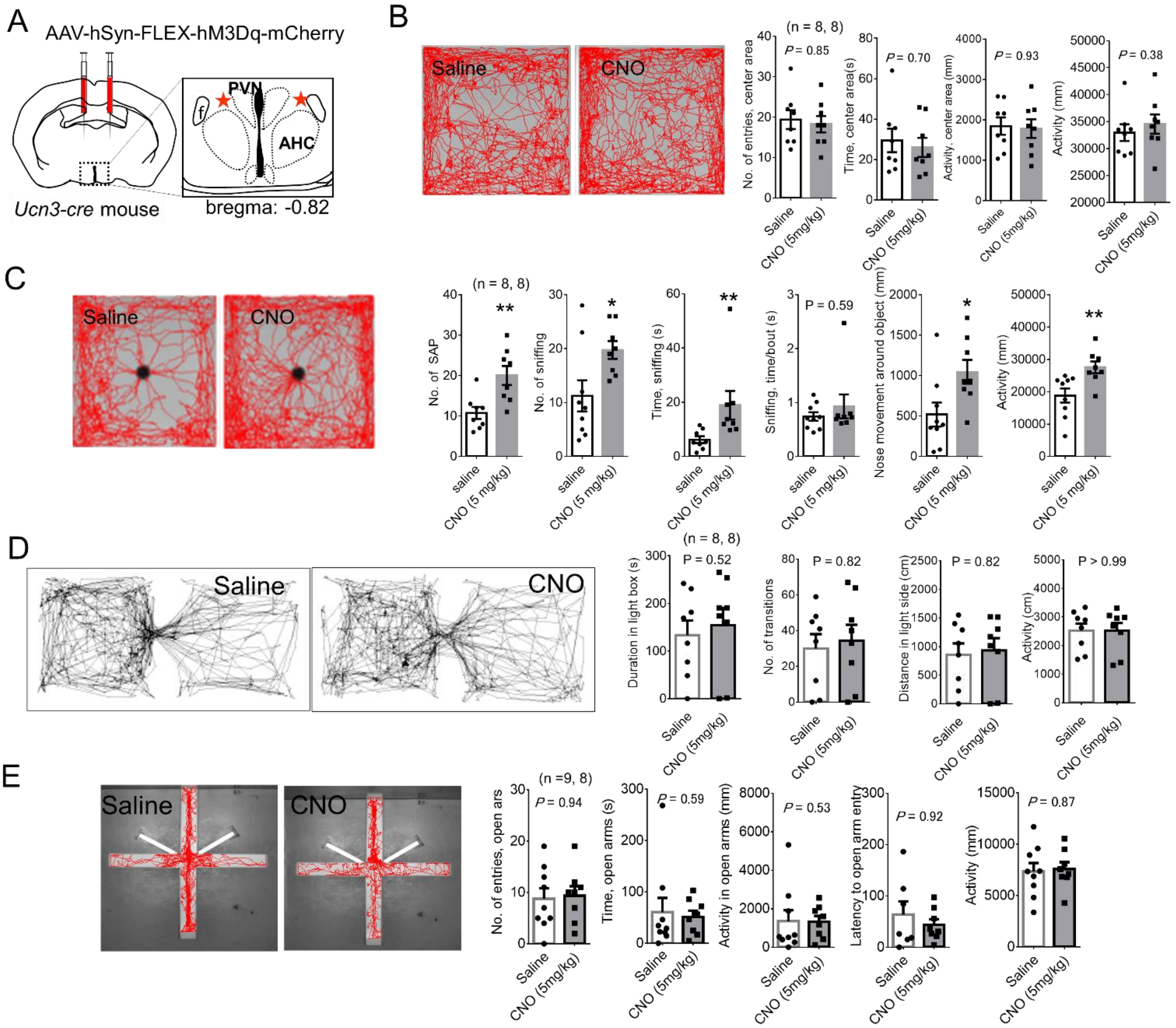

Figure S3 Behavioral effects of activation of PeFA Ucn3 neurons by hM3Dq DREADD with CNO (5 mg/kg mouse). Related to Figure 3.

(A)A schematic representation of Cre-dependent hM3Dq-mCherry AAV injection into the PeFA in *Ucn3-Cre* mice at -0.82 mm to the bregma.

(B)Open-field test (n = 8, 8). Representative images of body-center tracking in saline- (left) or CNO- (right, 5 mg/kg mouse) treated animal. Graphs show the number of entries into the center area (Mann-Whitney, U = 30, P = 0.85), duration of stay in the center area (Mann-Whitney, U =28, P = 0.70), activity in the center area (Mann-Whitney, U = 31, P = 0.93), and activity in a whole field (Mann-Whitney, U = 23, P = 0.38). Data are represented as mean ± SEM.

(C)Novel-object test (n = 8, 8). Representative images of nose-point tracking in saline- (left) or CNO-(right, 5 mg/kg mouse) treated animal. Graphs show the number of SAP (Mann-Whitney, U =7, **P < 0.01), the number of sniffing (Mann-Whitney, U = 14, *P < 0.05), time engaged in sniffing (Mann-Whitney, U = 4, **P < 0.01), nose movement around the object (Mann-Whitney, U = 12, *P < 0.05), time per sniffing bout (Mann-Whitney, U = 30, P = 0.59), and activity in a field (Mann-Whitney, U = 6, **P < 0.01). Data are represented as mean ± SEM.

(D)Light-dark box test (n = 8, 8). Representative images of body-center tracking in saline- (left) or CNO-(right, 5 mg/kg mouse) treated animal. Graphs show the duration of stay in light side (Mann-Whitney, U =25.5, P = 0.52), the number of transitions (Mann-Whitney, U = 29.5, P = 0.82), distance moved in light side (Mann-Whitney, U = 29.5, P = 0.82), and activity (Mann-Whitney, U = 32, P > 0.99). Data are represented as mean ± SEM.

(E)Elevated plus-maze test (n = 8, 8). Representative images of body-center tracking in saline- (left) or CNO-(right, 5 mg/kg mouse) treated animal. Graphs show the number of entries into open arms (Mann-Whitney, U =35, P = 0.94), the duration of stay in open arms (Mann-Whitney, U = 30, P = 0.59), activity in open arms (Mann-Whitney, U = 29, P = 0.53), latency to the first open arm entry (Mann-Whitney, U = 27, P = 0.92), and activity (Mann-Whitney, U = 34, P = 0.87). Data are represented as mean ± SEM.

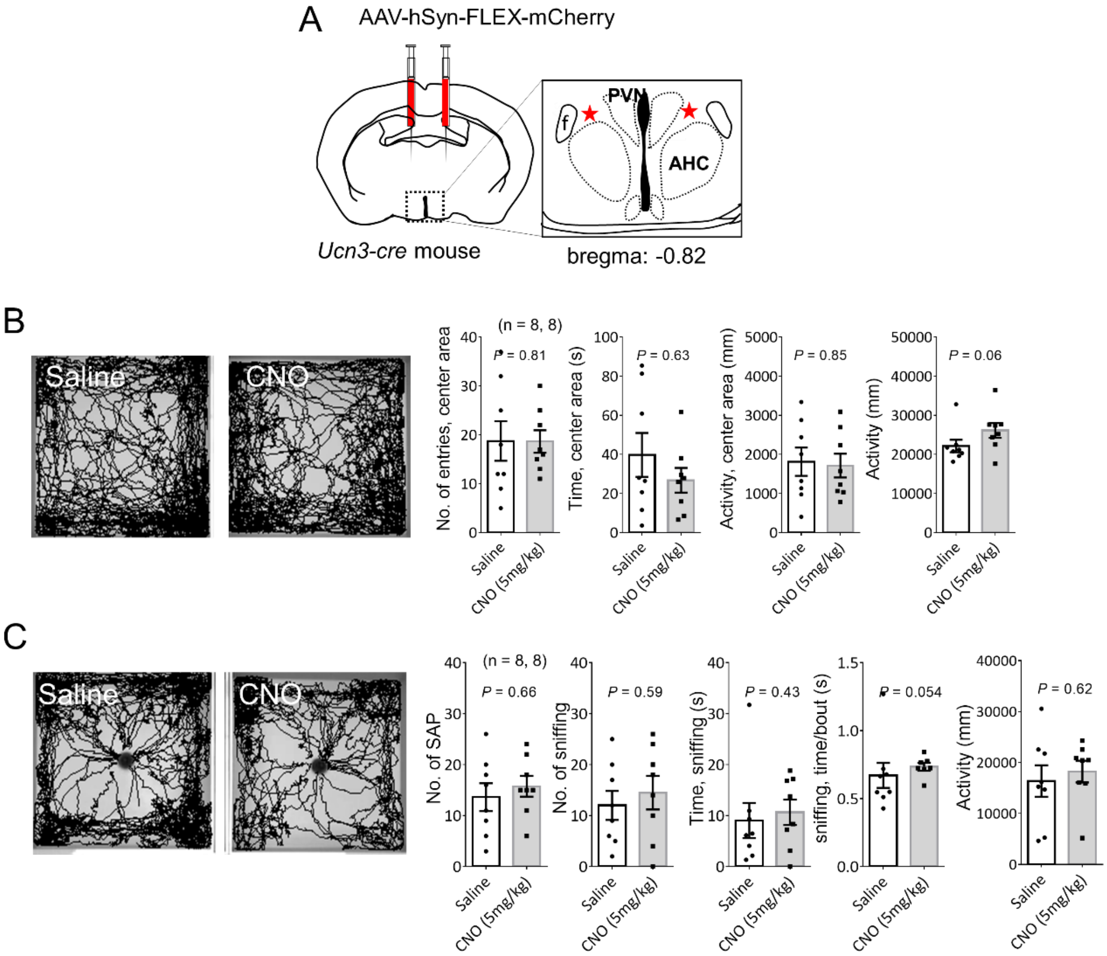

Figure S4 Effects of CNO (5 mg/kg mouse) on behaviors in *Ucn3-Cre* mice injected with AAV control vector of FLEX-mCherry. Related to Figure 3.

(A)A schematic representation of Cre-dependent mCherry AAV injection into the PeFA in *Ucn3-Cre* mice at -0.82 mm to the bregma.

(B)Open-field test (n = 8, 8). Representative images of body-center tracking in saline- (left) or CNO- (right, 5 mg/kg mouse) treated animal. Graphs show the number of entries into the center area (Mann-Whitney, U = 28.5, P = 0.74), duration of stay in the center area (Mann-Whitney, U =29, P = 0.78), activity in the center area (Mann-Whitney, U = 31, P = 0.93), and activity in a whole field (Mann-Whitney, U = 26, P = 0.56). Data are represented as mean $\pm$ SEM.

(C)Novel-object test (n = 8, 8). Representative images of nose-point tracking in saline- (left) or CNO-(right, 5 mg/kg mouse) treated animal. Graphs show the number of SAP (Mann-Whitney, U =27.5, P = 0.66), the number of sniffing (Mann-Whitney, U = 26.5, P = 0.59), time engaged in sniffing (Mann-Whitney, U = 24, P = 0.43), time per sniffing bout (Mann-Whitney, U = 11, P = 0.054), and activity in a field (Mann-Whitney, U = 27, P = 0.62). Data are represented as mean $\pm$ SEM.

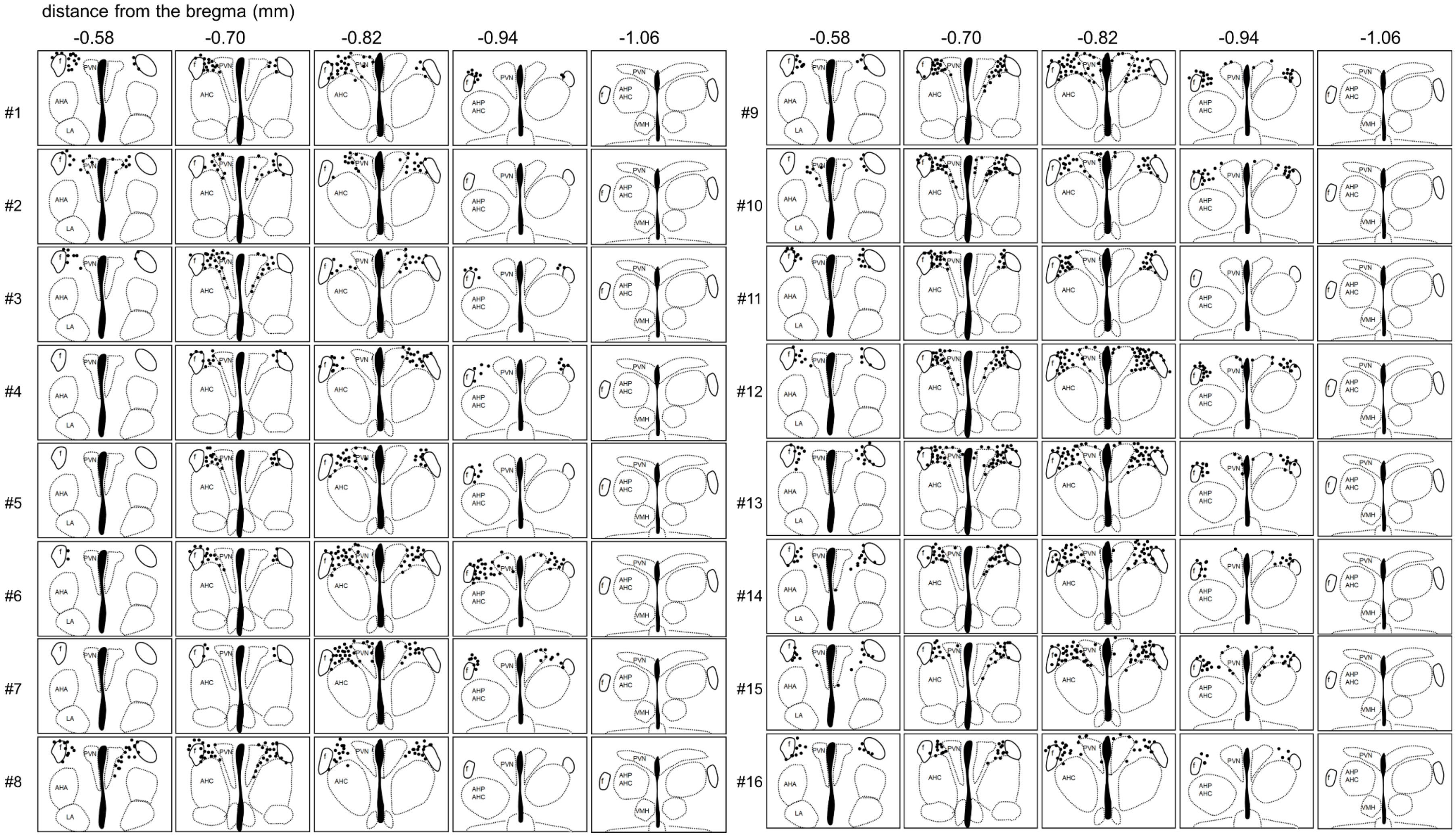

Figure S5 The locations of mCherry⁺ cells in AAV (hSyn-FLEX-hM3Dq-mCherry)- injected *Ucn3-Cre* mice used for behavioral testing. Related to Figure 3. The illustrations indicate the locations of mCherry+ cells (black dots) from 16 animals used for behavioral testing (8 animals from each group, 0.7 mg/kg CNO or saline group).

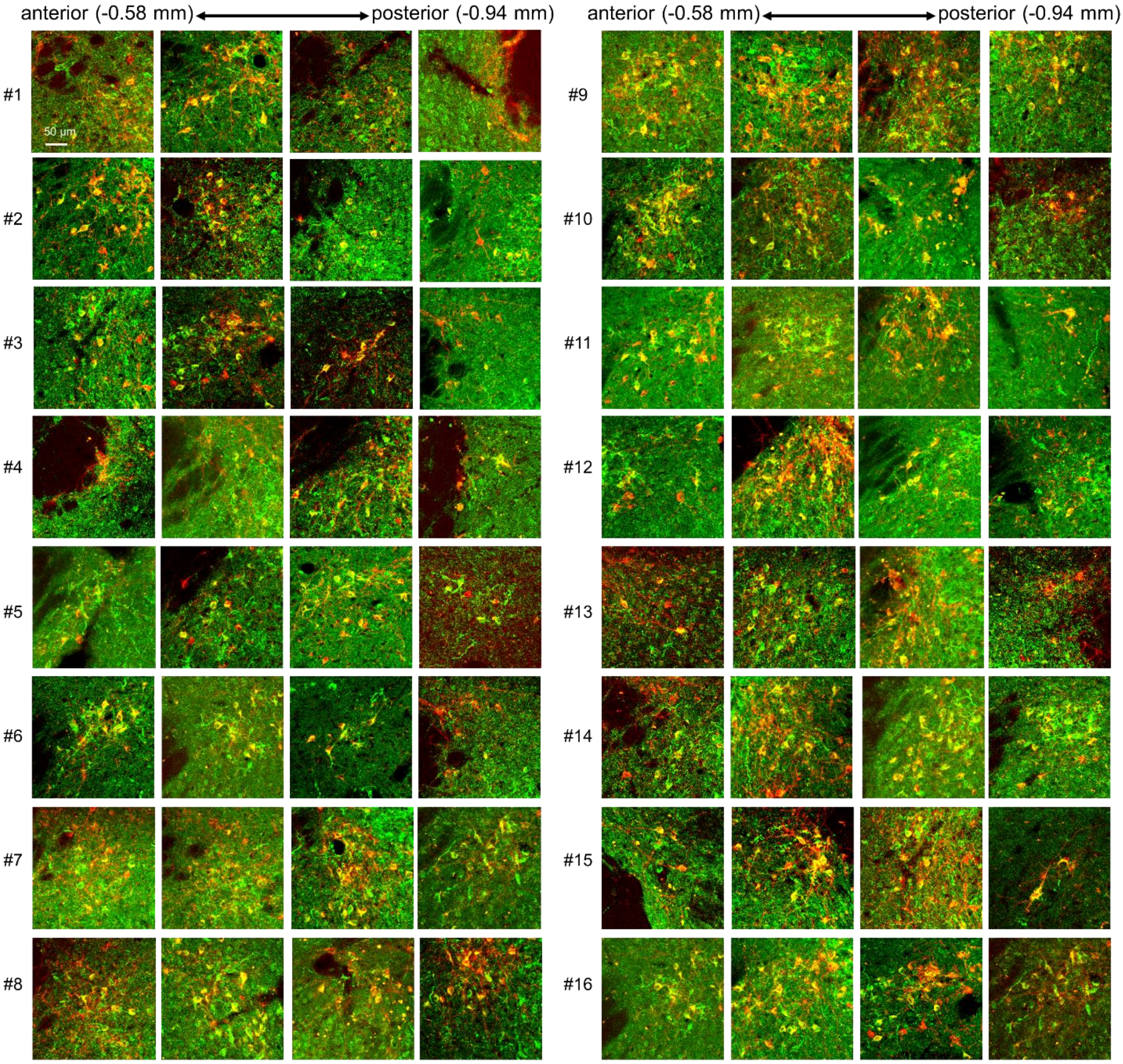

Figure S6 The colocalization of Ucn3⁺ cells and mCherry⁺ cells in AAV (hSyn-FLEX-hM3Dq-mCherry)-injected *Ucn3-Cre* mice used for behavioral testing. Related to Figure 3.

Fluorescent images of the overlay of Ucn3 (green) and mCherry (red) from 16 animals used for behavioral testing (8 animals from each group, 0.7 mg/kg CNO or saline). Quantification indicated 87.0 $\pm$ 1.68% of mCherry$^+$ cells were immunoreactive for Ucn3 (the mean $\pm$ SE, n = 16). Scale bar = 50 μm.

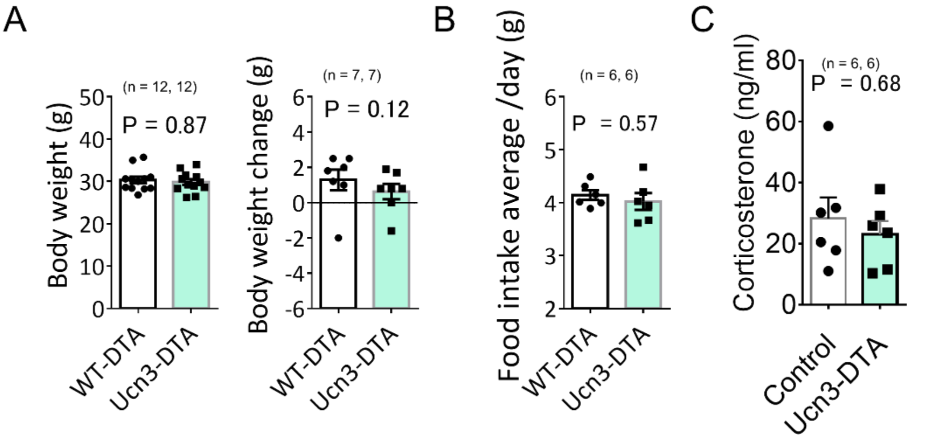

Figure S7 Body weight, food intake, and plasma corticosterone levels in Ucn3-DTA mice. Related to Figure 3.

(A)Body weight and its change 4 weeks after viral injection of Cre-dependent AAV encoding DTA-mCherry in wildtype (WT-DTA) and Ucn3-Cre (Ucn3-DTA) mice. (body weight, Mann-Whitney, U = 69, P = 0.87; body weight change, Mann-Whitney, U = 12, P = 0.12). Data are represented as mean ± SEM.

(B)The daily food intake average for 2 weeks from 2 weeks after viral injection (Mann-Whitney, U = 14, O = 0.57). Data are represented as mean ± SEM.

(C)Plasma corticosterone levels in WT-DTA and Ucn3-DTA (Mann-Whitney, U = 15, P = 0.68). Data are represented as mean ± SEM.

Transparent Methods

All procedures for animal experiments were approved by the Animal Care Committee of Nara Medical University or Azabu University (#180316-6) and were performed according to the NIH Guidelines and the Guidelines for Proper Conduct of Animal Experiments published by Science Council of Japan. C57BL/6 and *Ucn3-cre* male mice were used; the latter were purchased from MMRRC (Stock #: 032078-UCD). All mice used in the experiments were 8–24-weeks old and were housed under standard laboratory conditions with ad libitum access to food and water (23°C, 55% humidity in a room, and a 12 h light–dark cycle: lights-on at 8:00 a.m.). For all experiments, mice were age-matched and randomly assigned to experimental groups to exclude a biased distribution of animals. Mice used for behavioral testing were littermates.

Viral Vectors and Stereotaxic Surgery

AAV vectors for DREADD (hSyn-FLEX-hM3Dq-mCherry, DJ, 1×10^{13} copies/ml), its control vector (hSyn-FLEX-mCherry, DJ, 1×10^{13} copies/ml), and the targeted cell death method with DTA (CMV-FLEX-mCherry/Diphtheria toxin A fragment, AAV-10, 4×10^{12} copies/ml), were produced using the AAV Helper-Free System (Agilent Technologies, Inc., Santa Clara, CA, USA) in accordance with a previous work (Inutsuka et al., 2014). Briefly, HEK293 cells were transfected with a pAAV vector plasmid that included a gene of interest, pHelper, and pAAV-RC provided by Penn Vector Core using a standard calcium phosphate method. Three days later, the transfected cells were collected and suspended in artificial CSF (124 mM NaCl, 3 mM KCl, 26 mM NaHCO3, 2 mM CaCl2, 1 mM MgSO4, 1.25 mM KH2PO4, 10 mM D - Glucose). After 4 freeze-thaw cycles, the cell lysate was treated with benzonase nuclease (Merck, Darmstadt, Germany) at 45ºC for 15 min, and centrifuged 2 times at 16,000 g for 10 min. The supernatant was used as the virus-containing solution. To measure the titer of purified virus dissolved in artificial CSF, quantitative PCR was performed; the virus was stored at –80ºC in aliquots before use. The pAAV-hSyn-FLEX-hM3Dq-mCherry plasmid was purchased from Addgene (ID: 44361). An AAV vector for fiber photometry (syn-FLEX-jGCaMP7s-WPRE, 104491-AAV9, 3×10^{13} copies/ml) was purchased from Addgene (Watertown, MA).

Stereotaxic AAV injection was performed under anesthesia with 2% isoflurane, using an automated injector (Narishige, Tokyo, Japan) connected to a Neuros Syringe (Hamilton, Reno, NV). The AAV vectors (250 nl for DREADD experiment and 600 nl for fiber photometric experiment) were injected into the PeFA according to the mouse brain atlas (stereotaxic coordinate: AP = -0.82 mm, ML = ± 0.47 mm, DV = 4.5 mm from the dura matter) at a flow rate of 40–100 nl/min. For fiber photometry experiments, an optical fiber (Doric Lenses;

Québec, QC; diameter, 400 µm) was implanted at the same coordinates. After surgery, mice were singly housed for 4 weeks and then used for experiments. The accuracy of the injection site was checked in all mice subjected to surgery. Colchicine (4 mg/ml, 500 nl) was injected into the lateral or the fourth ventricles; 4 days after colchicine injection, mice were sacrificed and used for immunohistochemical staining.

Immunohistochemistry

Mice were anesthetized with sodium pentobarbital (100 mg/kg) and perfused transcardially with phosphate-buffered saline (PBS) followed by 4% paraformaldehyde in 0.1 M phosphate buffer. Brains were post-fixed for 6–16 h at 4°C. Fifty-micrometer-thick sections were cut by a vibratome (Microslicer; Dosaka, Kyoto, Japan), before immersion in 25 mM glycine in PBS, 0.3% Triton X-100 in PBS (PBST), and blocking with 5% normal horse serum in PBST for 2 h. Sections were incubated with primary antibodies diluted in the same blocking solution for 2 days at 4°C. Primary antibodies used were guinea pig anti-c-Fos (1:1000, Synaptic System, Goettingen, Germany), rabbit anti-urocortin 3 (1:200, Yanaihara, Shizuoka, Japan), mouse anti-Enk (1:500, Novus Biologicals) and rat anti-mCherry (1:200, ThermoFisher Scientific, Yokohama, Japan). After three washes with PBS, the sections were incubated with species-specific secondary antibodies conjugated to Alexa Fluor 488 or 594 (ThermoFisher Scientific) for 2 h. After three washes with PBS, the sections were mounted on glass slides, and coverslipped with Vectashield containing 4', 6-diamidino-2-phenylindole dihydrochloride (DAPI; Vector, Burlingame, CA). Observation and acquisition of fluorescent images were performed with a confocal microscope (FluoView 1000, Olympus, Tokyo, Japan) and cell counting and image analysis were carried out using Metamorph software (Molecular Devices, San Jose, CA).

c-Fos Expression Analysis

Animals were singly housed and habituated to the housing for at least 10 days. Some animals were housed with a novel object, a plastic ball with a 4-cm diameter, during this period to become familiar with the object. One of the following stimuli was given to the mice at 10:00 am in their home cages or new empty cages, 2-(Methylthio)-2-thiazoline (2MT, TCI chemicals, Tokyo, Japan), eugenol (TCI chemicals), a conspecific male mouse as an intruder, novel object, familiar object, restraint stress for 2 h, and pain by subcutaneous injection of formalin solution (10 µl) into the hind paw. Pain induction was confirmed by observing nocifensive behaviors, such as licking and flinching/shaking of the paws, according to a previous report (Hunskaar and Hole 1987). Two hours after these stimuli, mice were sacrificed and fixed for

immunohistochemical staining, as described above. Cell counting was performed on fluorescent images by using Metamorph software.

Fiber Photometry

In the fiber photometry experiments, we used two objects, a LEGO block and a ping-pong ball. One item was placed in the animal's home cage for more than 2 weeks before the recording experiments and was thus designated as the familiar object. The other was designated as the novel object. Associations between objects and familiarity were counterbalanced across animals. On the day of the experiment, a patch cord was connected to the implanted fiber, under brief anesthesia, and the animal was transferred to a test cage (24 × 17 × 13 cm). After a 30-min habituation, the recording experiment started; calcium signals were monitored through the implanted fiber while the animal was sequentially presented with a familiar object, a novel object, and the familiar object again. Each object was initially placed far from the animal, and the animal was allowed to interact with the object for 5 min. If there were not sufficient interactions with the object in the first 5 min, the behavioral block was extended for an additional 5 min. Two excitation lights (465- and 405-nm LED lights that were amplitude-modulated by 225- and 525-Hz sine waves, respectively) were used (Doric Lenses; DFG-2022F, Owon), which produced GCaMP and isosbestic signals. The emitted light was collected by a photodetector (Model 2151, Newport Corp., Irvine, CA), digitized with a sampling rate of 5,000 Hz (USB-6212 BNC, National Instruments, Austin, TX), and stored on a computer. The experiments were videotaped from the top (frame rate, 30 Hz; DMK23U618, The Imaging Source, Taipei, Taiwan). Data acquisition was controlled by custom-made Bonsai workflow software (Lopes et al., 2015). The signals were demodulated and normalized offline using a Matlab (MathWorks) script. The demodulated signals were detrended and scaled such that the standard deviations of the signals matched. The ratio of GCaMP7s signals with isosbestic signals was defined as the normalized fluorescent signal (Fn). For the peri-event time histogram analysis, we calculated $\frac{\Delta F}{F_0} = \frac{F_1 - F_0}{F_0}$, where F_0 indicates average normalized fluorescent signals during 5 s before a behavioral event, and F_1 indicates the normalized fluorescent signals at any given time point. We used the grand average across all animals for data visualization and analyses. The behavioral events were manually annotated using the Boris software (Friard and Gamba, 2016).

SAP behavior was defined as the posture that animals lower the back, stretch the neck, and elongate the body toward an object whilst either standing still or moving forward. SAP behavior was further classified into two types based on whether the mouse's nose was in close proximity to the object (≦1 cm, SAP-sniffing) or not (1 cm <, SAP-nonsniffing). The

approach without SAP behavior was defined as approaching close (≦1 cm) to an object without performing SAP behavior.

Behavioral Testing

All behavioral tests were performed during the light phase from 9:30 to 14:00. Mice were transferred to a test room at least 30 min before commencing testing. Mice were subjected to, at most, one test per day, except for the open-field and novel-object tests, which were performed serially. TopScan LITE software (CleverSys Inc., Reston, VA) was used for automated behavioral analysis. When performing visual observations, experimenters were blinded to animal groups. CNO was purchased from Abcam (Cambridge, UK). Mice used for behavioral testing were littermates.

Open-Field and Novel-Object Tests

For DREADD experiments, CNO (0.7 mg/kg or 5 mg/kg mouse) was intraperitoneally injected 15 min before behavioral testing. Mice were allowed to move freely in an open field (40 × 40 × 40 cm) under 100 lux brightness for 10 min before being taken out of the field, and immediately returned to their home-cage. After cleaning the open field, a novel object (house-shaped ceramic toy: 5 cm-height, the diameter of the edge of the roof was 4 cm) was placed in the center of the field. Mice were again allowed to explore the object for 10 min. In the open-field test, a center area was defined as a 13.3 × 13.3 cm^2 region in the center of the field. Animal movement was analyzed using TopScan LITE by tracking the center point of the body. The numbers of entries into the center area, time spent in the area, and locomotor activity were measured.

In the novel-object test, both the point of the nose and the center of the body were tracked using the same software. The number of SAP behaviors was counted by experimenters blinded to animal groups and sniffing and locomotor activity were measured using the software.

Novel-Object Test in the Home-Cage

A recording apparatus (O’Hara & Co., Ltd., Tokyo, Japan) was set in the home cage. The brightness in the cage was adjusted to 5 lux and mice were given 15 min to habituate to the apparatus. Activity changes in the home-cage without a stainless mesh lid was measured for 15 min using TopScan LITE by tracking a center point of the body. A 15-ml plastic tube, a coiled piece of barbed wire, or a 5-cm-long electrified shock prod with a 0.8 cm-diameter and a conducting wire coiled at a 1.6-mm-interval (O’Hara & Co., Ltd.) was used as the novel object. The prod emitted an electric current (0.5 mA) when a moist object, such as an animal’s

nose or mouth/teeth, touched the wire. Mice were allowed to explore the object freely for 10 min. Gnawing, SAP, and shocking were observed and counted by experimenters blinded to the animal group allocation. Nose entry into the object area (1-cm around the object) and activity were analyzed using TopScan LITE by tracking the nose and the center point of the body.

Marble-Burying Test

For DREADD experiments, CNO (0.7 mg/kg or 5 mg/kg mouse) was injected 15 min before behavioral testing. The test was performed as described before (Deacon, 2006). Briefly, mice were allowed to move freely for 30 min in a standard laboratory cage that contained sufficient bedding material (4 cm-height from the floor) and 24 (4 × 6) glass marbles aligned on the surface of the material. The numbers of buried marbles that were covered with bedding material over more than two-thirds of their surface were counted.

Single-Object Burying Test

CNO (5 mg/kg mouse) was injected into mice 15 min before testing. Mice were allowed to move freely in a new cage containing fresh bedding material (4-cm height from the floor) without objects for 10 min before being taken out of the cage, and immediately returned to their home-cage. In the meantime, an experimenter placed a novel object (a plastic ball with a 4-cm diameter or a column-shaped plastic socket with a 3-cm diameter and 5.5-cm height) in the cage. Mice were allowed to explore the object freely for 10 min. Burying and SAP were measured by experimenters blinded to animal groups and sniffing and locomotor activity were measured by TopScan LITE by tracking the nose point and the center point of the body, respectively.

Light–Dark Box Test

CNO (5 mg/kg mouse) was injected into mice 15 min before testing. The light–dark box test was performed according to a previous report (Takao and Miyakawa, 2006) and the apparatus was purchased from O'Hara & Co., Ltd. (Each box size was 20 × 20 × 25 cm). The brightness of the dark box was adjusted to 5 lux and that of the light box was set to 600 lux. Mice were introduced into the dark box at the start of the test and were allowed to freely move and transit between the two boxes through a small doorway for 15 min. Measured parameters were included the time spent in the light box, distance moved in the light box, the number of transitions, and a whole locomotor activity.

Elevated-Plus Maze Test

CNO (5 mg/kg mouse) was injected into mice 15 min before testing. The elevated plus-maze test was performed according to a previous report (Komada et al., 2008). Briefly, the apparatus was purchased from O'Hara & Co., Ltd. The apparatus is made with two open arms (25 × 5 × 0.5 cm) and two closed arms (25 × 5 × 16 cm), with a center platform (5 × 5 × 0.5 cm) and is elevated 50 cm above the floor. A mouse was placed in the center area of the maze with its head directed toward a closed arm and was allowed to explore the maze freely for 5 min. The number of entries into each arm and the time spent in the open arms were measured using TopScan LITE by tracking the center point of the body.

Corticosterone Assay

Blood samples were collected after mice were decapitated between 10:00 and 11:00 a.m. and plasma was prepared by centrifugation at 2,000 × *g* for 15 min at 4°C. The samples were kept at -85°C until the day of the assay. Corticosterone concentrations were measured using a Corticosterone EIA kit (Yanaihara Institute, Fujinomiya, Japan) according to the manufacturer's instructions.

QUANTIFICATION AND STATISTICAL ANALYSIS

All data were expressed as the mean ± SEM. GraphPad Prism 6 was used to analyze data and plot figures. Mann-Whitney U, Kruskal-Wallis, and Wilcoxon singed-rank test were used for statistical analysis. When the results of Kruskal-Wallis were significant, Dunnett's test was used for a post-hoc multiple comparisons test. Statistical data are provided in the figures and legends. $P < 0.05$ was considered statistically significant.

Supplemental References

Deacon, R.M. (2006). Digging and marble burying in mice: simple methods for in vivo identification of biological impacts. Nature protocols *1*, 122-124.

Friard, O., and Gamba, M. (2016). BORIS: a free, versatile open-source event-logging software for video/audio coding and live observations. Methods Ecol Evol *7*, 1325-1330.

Inutsuka, A., Inui, A., Tabuchi, S., Tsunematsu, T., Lazarus, M., and Yamanaka, A. (2014). Concurrent and robust regulation of feeding behaviors and metabolism by orexin neurons. Neuropharmacology *85*, 451-460.

Komada, M., Takao, K., and Miyakawa, T. (2008). Elevated plus maze for mice. Journal of visualized experiments : JoVE.

Lopes, G., Bonacchi, N., Frazao, J., Neto, J.P., Atallah, B.V., Soares, S., Moreira, L., Matias, S., Itskov, P.M., Correia, P.A.*, et al.* (2015). Bonsai: an event-based framework for processing and controlling data streams. Front Neuroinform *9*, 7.

Takao, K., and Miyakawa, T. (2006). Light/dark transition test for mice. Journal of visualized experiments : JoVE, 104.
